# NiCo_2_O_4_ Nano-/Microstructures as High-Performance Biosensors: A Review

**DOI:** 10.1007/s40820-020-00462-w

**Published:** 2020-06-08

**Authors:** Rajesh Kumar

**Affiliations:** Department of Chemistry, Jagdish Chandra DAV College, Dasuya, Distt. Hoshiarpur, 144205 Punjab India

**Keywords:** Nano-/micro-structured, Spinel NiCo_2_O_4_, Synthetic methods, Modified electrodes, Electrochemical biosensors

## Abstract

Non-enzymatic biosensors based on mixed transition metal oxides are deemed as the most promising devices due to their high sensitivity, selectivity, wide concentration range, low detection limits, and excellent recyclability. Spinel NiCo_2_O_4_ mixed oxides have drawn considerable attention recently due to their outstanding advantages including large specific surface area, high permeability, short electron, and ion diffusion pathways. Because of the rapid development of non-enzyme biosensors, the current state of methods for synthesis of pure and composite/hybrid NiCo_2_O_4_ materials and their subsequent electrochemical biosensing applications are systematically and comprehensively reviewed herein. Comparative analysis reveals better electrochemical sensing of bioanalytes by one-dimensional and two-dimensional NiCo_2_O_4_ nano-/microstructures than other morphologies. Better biosensing efficiency of NiCo_2_O_4_ as compared to corresponding individual metal oxides, viz. NiO and Co_3_O_4_, is attributed to the close intrinsic-state redox couples of Ni^3+^/Ni^2+^ (0.58 V/0.49 V) and Co^3+^/Co^2+^ (0.53 V/0.51 V). Biosensing performance of NiCo_2_O_4_ is also significantly improved by making the composites of NiCo_2_O_4_ with conducting carbonaceous materials like graphene, reduced graphene oxide, carbon nanotubes (single and multi-walled), carbon nanofibers; conducting polymers like polypyrrole (PPy), polyaniline (PANI); metal oxides NiO, Co_3_O_4_, SnO_2_, MnO_2_; and metals like Au, Pd, etc. Various factors affecting the morphologies and biosensing parameters of the nano-/micro-structured NiCo_2_O_4_ are also highlighted. Finally, some drawbacks and future perspectives related to this promising field are outlined.

## Highlights

- Various synthetic methods for the synthesis of NiCo_2_O_4_ nano-/microstructures in bare, doped, and composite/hybrid forms are reviewed.
- Currents status and development prospects of NiCo_2_O_4_ nano-/microstructure-based electrochemical biosensors for bioanalytes such as glucose, urea, and H_2_O_2_, along with condition governing the electrochemical biosensor parameters, are summarized.
- Also provide an insight into the key challenges and future perspectives about point-of-care monitoring of bioanalytes using NiCo_2_O_4_ nano-/microstructure-based biosensors.

## Graphical abstract

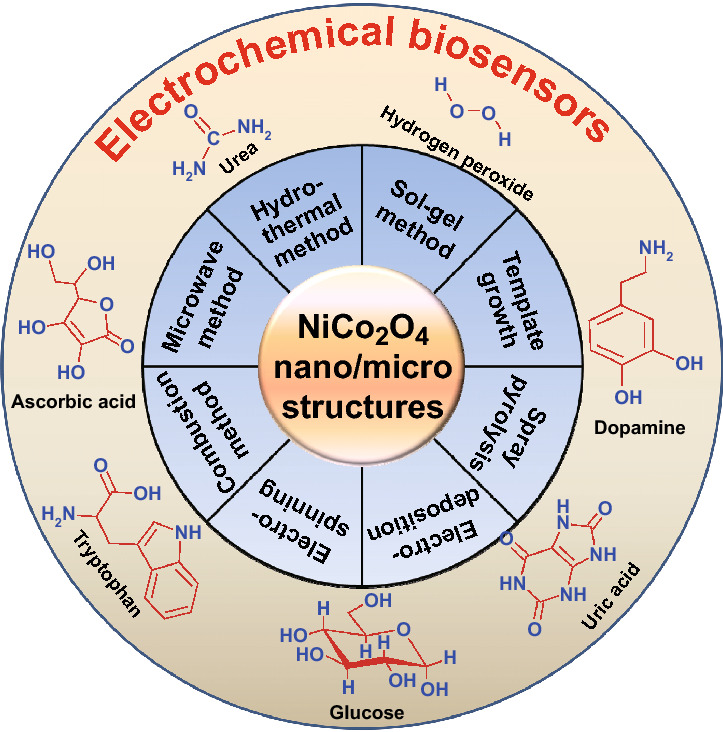

## Introduction

Recently, spinel single-phase binary metal oxides containing two metal cations such as manganese cobaltate (MnCo_2_O_4_) [1], zinc cobaltate (ZnCo_2_O_4_) [2, 3], nickel ferrite (NiFe_2_O_4_) [4], copper manganate (CuMn_2_O_4_) [5], copper cobaltate (CuCo_2_O_4_) [6], cobalt manganate (CoMn_2_O_4_) [7], nickel cobaltate (NiCo_2_O_4_) [8] have attracted widespread attention from researchers worldwide due to their invariably better electrochemical properties as compared to individual metal oxides or a mixture of metal oxides. The excellent electrochemical performances of these single-phase binary metal oxides are attributed to the synergetic effects of properties of the individual metal oxide components [9]. Among various such single-phase binary metal oxides, NiCo_2_O_4_ is considered to be the best one as it possesses at least two times higher electronic conductivity as compared to corresponding individual metal oxides, viz. NiO and Co_3_O_4_ along with intrinsic-state redox couples of Ni^3+^/Ni^2+^ (0.58 V/0.49 V) and Co^3+^/Co^2+^ (0.53 V/0.51 V) [10–12]. Other key features are the exhibition of variable but sufficiently stable oxidation states by Ni (Ni^2+^, Ni^3+^) and Co (Co^2+^, Co^3+^, Co^4+^) and very high conductivity of 500 S cm^−1^ [13, 14].

Many transition metals, rare earth metals, non-metal-doped NiCo_2_O_4_, and conjugated polymer-modified NiCo_2_O_4_ materials have been reported in the literature with versatile applications. N- and P-doped NiCo_2_O_4_ with oxygen vacancies have been explored for electrochemical performance for supercapacitors, electro-catalyst for O_2_ and H_2_ evolution reaction [15–18], and anodic material for lithium-ion batteries [19]. Lin et al. [20] explored S-doped NiCo_2_O_4_ nanosheet arrays as the efficient and bifunctional electrode for overall water-splitting reactions. Compared with non-metal-doped NiCo_2_O_4_, transition metal and rare earth metal-doped NiCo_2_O_4_ are considered superior due to the latter’s excellent electrical conductivity. Zn- and Fe-doped NiCo_2_O_4_ showed electrocatalytic properties for oxygen evolution reactions and remarkable capacitive properties in asymmetric supercapacitors [21–23]. Ma et al. [24] synthesized highly porous hierarchical spinel Mn-doped NiCo_2_O_4_ nanosheets for high-performance anodes in lithium-ion batteries. Xia et al. [25] used Au–NiCo_2_O_4_ nanomaterials supported on 3D hierarchical porous graphene-like material as electro-catalyst for oxygen evolution reaction. Among the rare earth metal oxides, CeO_2_ is reported to be an excellent dopant for NiCo_2_O_4_ nanomaterials [26, 27]. Carbonaceous and polymer composite/hybrid NiCo_2_O_4_ nano-/microstructures are also found suitable for their potential applications in supercapacitors [28], fuel cells [29], Li-ion batteries [30], electro-catalyst for oxygen reduction reaction and oxygen evolution reaction [31], photo-detector [32], optoelectronic devices [33], perovskite solar cells [34], gas sensors [35–37] and biosensors [38, 39].

**Fig. 1:**
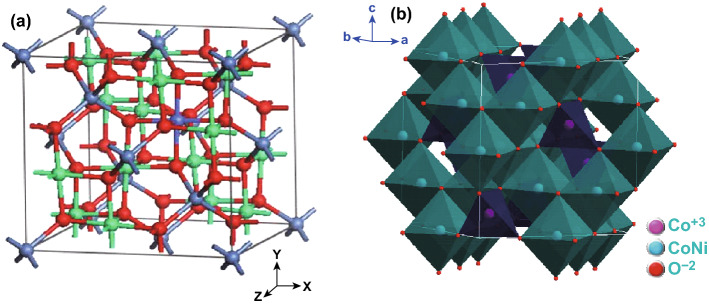
**a** Crystal structure of NiCo_2_O_4_. Reproduced with permission from Ref. [41]. Copyright © 2014 Elsevier B.V. **b** NiCo_2_O_4_ cubic spinel. Reproduced with permission from Ref. [42]. Copyright © 2013 American Chemical Society

Facile, low-cost and eco-friendly synthetic methods lead to varieties of low dimensional nano-/micro-structured morphologies with excellent porosity and specifically large surface area, opportunities to synthesize composite/hybrid and ease of electrode fabrications for end-user applications. Spinel NiCo_2_O_4_ is a p-type semiconductor in which Ni occupies octahedral sites while Co is distributed in both octahedral and tetrahedral sites [13] (Fig. 1a, b). It shows a face-centered cubic arrangement and belongs to Fd3m space group with lattice constant *a*_o_ = 8.269 Å [40].

Electrochemical sensing through miniaturized sensors based on nano-/micro-structured materials has taken over the conventional, expensive, laborious sensing techniques like lateral flow immunoassay, liquid chromatography, capillary electrophoresis, enzyme-linked immunosorbent assay, chemiluminescence, sequential injection analysis, gas chromatography–mass spectrometry and fluorescent methods [43–48]. Electrochemical biosensors can be categorized into amperometric and potentiometric sensors [49]. The amperometric biosensing involves a change in current response due to electrochemical redox reactions of the analytes when a potential is applied between the working and reference electrodes while the potentiometric biosensing makes use of ion-selective electrodes to transduce the biological reactions into a measurable electrical signal [43, 50].

Among the main classes of biosensors, the non-enzymatic biosensor is considered to be better, faster, and more convenient as compared to an enzymatic biosensor that involves complicated and multi-step enzyme immobilization processes and high specificity of the enzymes. Also, due to pH and temperature sensitiveness, the enzyme-based biosensors are highly unstable as enzymes undergo denaturation leading to biological inactivity beyond physiological conditions [51–53]. Nanomaterials not only provide high-density catalytic sites for the electro-oxidation or electro-reduction in the biomarkers but also provide large surface area for adsorption of biomarkers and facilitate an appropriate path for electron transport for electrochemical activity [54–56]. Since the crucial part in electrochemical biosensors is the modified electrode, much attention has been devoted to modulate the electrocatalytic behavior of the NiCo_2_O_4_ as electron mediator by engineering its composition, structure, specific surface area, and redox properties.

To date, many reviews have been reported for the applications of NiCo_2_O_4_ nano-/micro-structured materials including Li-ion batteries [10], supercapacitors [11, 57], fuel cells [58], and electro-catalyst for oxygen reduction, oxygen and hydrogen evolution reactions [59, 60]. The applications of the NiCo_2_O_4_-based non-enzymatic biosensors are aimed not only at the extension of the spectrum of target bioanalytes but also at the improvement in the biosensor performance in terms of sensitivity, selectivity, detection limits, long-term stability as well as reusability. Many new synthetic strategies and techniques have been developed for the fabrication of NiCo_2_O_4_-based non-enzymatic biosensors, but they are rarely summarized. Hence, it is an appropriate time to go through the periodical progress of NiCo_2_O_4_-based non-enzymatic biosensors. This review covers the crystal structure of the spinel NiCo_2_O_4_, various synthetic strategies employed for the synthesis of nano-/micro-structured NiCo_2_O_4_, electrochemical biosensing toward biomarkers such as glucose, H_2_O_2_, and urea, through the fabrication of modified electrodes. Various factors affecting the morphologies and biosensing parameters of the nano-/micro-structured NiCo_2_O_4_ are also reviewed.

## General Biosensing Mechanism

Two types of strategies are generally involved in the electrochemical biosensing of biomarkers, i.e., enzyme based and enzyme-free [61, 62]. An enzymatic biosensor operates on three main components which include sensitive recognition element, signal transducer element, and data evaluation component [63–66]. Enzymes, antibodies, and nucleic acid are generally used as recognition components. Glucose oxidase and glucose dehydrogenase for glucose [67, 68], horseradish peroxidase for H_2_O_2_ [69], urease for urea [70], laccase and polyphenol oxidase for rutin [71], tryptophan oxidase for tryptophan [72], etc. act as sensitive recognition elements. The function of the signal transducer is to convert chemical changes into detectable and readable electronic signals which are finally transferred to the data evaluation component. Recent developments in the field of nanotechnology and nanoscience reveal the excellent efficiencies of the nanostructured materials as signal transducers. Biosensors based on nanostructured materials as artificial bioreceptors are used for early detection and diagnosis of diseases through the estimation of the levels of biomarkers [73–75]. The signal transducer behavior of the nanomaterials mainly depends upon the electrochemical redox properties, surface-to-volume ratio, crystal structure and phase, morphology, and the presence of some other conducting matrices along with the nanostructured materials [76–78]. In contrast, in enzyme-free biosensors, nanostructured materials are used as signal transducers as well as sensitive recognition elements.

**Fig. 2:**
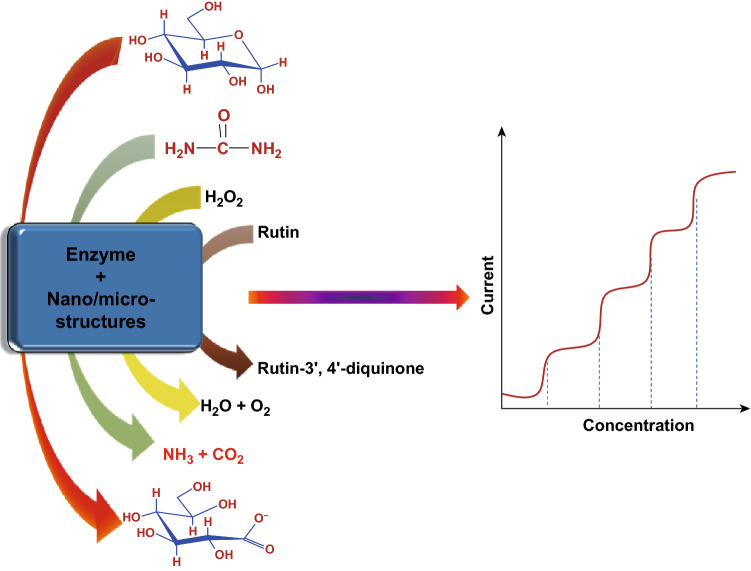
Proposed biosensing mechanism of nano-/microstructures

Electrochemical biosensors are mainly based on the output electrical signals changes incurred from either the oxidation or the reduction of the target bioanalyte on the surface of the transducer (Fig. 2) [79–81]. These redox reactions are catalyzed by signal transducer enzymes and nanostructured materials in enzyme-based and enzyme-free biosensors, respectively. The strength of the electrical signals is significantly affected by the concentrations of target bioanalytes, temperature, pH, and the presence of the interfering species [82–85].

## Synthesis of Nano-/Micro-Structured NiCo_2_O_4_

### Hydrothermal/Solvothermal Method

Hydrothermal synthesis involves heterogeneous reactions in an aqueous medium within a temperature range of 100–200 °C and high pressure. To achieve these conditions, the reaction is usually carried out in Teflon-lined sealed steel autoclaves. Alkali metal hydroxide or NH_3_ is added to convert the precursor metal salts into their respective hydroxides at basic pH conditions [86, 87]. An initial nucleation phase is followed by the directed crystal growth along appropriate crystal planes. The morphology, surface, and the structural features of the materials synthesized through hydrothermal method depend upon the conditions like temperature, pH of the solution, concentration of the precursor, nature of the solvent, and the presence of the templates [88]. NiCo_2_O_4_ nano-/microstructures of various shapes and morphologies have been prepared hydrothermally. Nano-/micro-structured NiCo_2_O_4_ of morphologies such as urchin shaped [89], coral-like [90], core–ring-structured nanoplatelets [91], porous coral-like nanospheres [36], hollow nanospheres [92], nanospheres [93], urchin-like spheres [94], mesoporous nanoparticles [95], mesoporous nanoneedles [96, 97], 3D network-like mesoporous nanostructures [98], 3D hierarchical tremella-like, flower-like, urchin-like and pine needle-like [99], nanoflakes [100], nanowalls [101], etc. are reported.

**Fig. 3:**
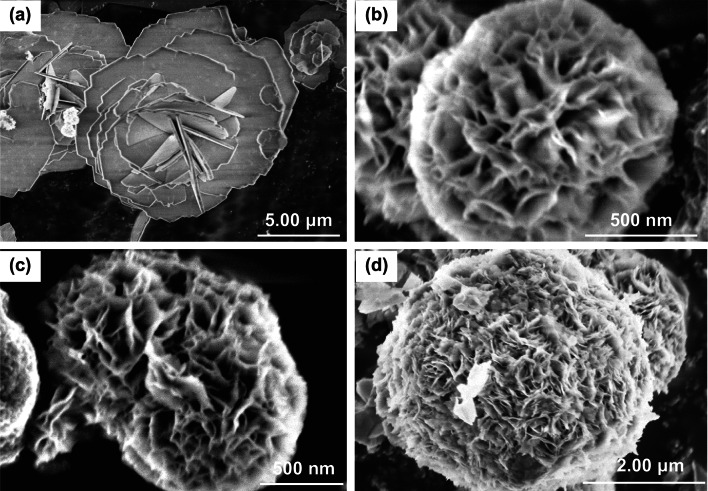
FESEM image of NiCo_2_O_4_ samples using water: ethanol ratios **a** 1:0, **b** 3:1, **c** 1:1, and **d** 1:3. Reproduced with permission from Ref. [109]. Copyright © 2017 Elsevier B.V.

Ni and Co precursor salt solutions with molar atomic ratio of 1:2 are taken during hydrothermal growth since Ni and Co atoms are present in the 1:2 atomic ratio. Liu et al. [94] used 1 mmol Ni(NO_3_)_2_·6H_2_O and 2 mmol Co(NO_3_)_2_·6H_2_O solution to prepare urchin-like NiCo_2_O_4_ spheres. Yang et al. [102] mixed 1 mmol of Ni(CH_3_COO)_2_·4H_2_O and 2 mmol of Co(CH_3_COO)_2_·4H_2_O for the preparation of NiCo_2_O_4_ nanospheres. Yu et al. [96] used 0.5 mmol Ni(NO_3_)_2_·6H_2_O, 1 mmol Co(NO_3_)_2_·6H_2_O for the synthesis of NiCo_2_O_4_ mesoporous nanoneedles. Zhu et al. [98] mixed 0.225 mmol of Ni(CH_3_COO)_2_·4H_2_O and 0.45 mmol of Co(CH_3_COO)_2_·4H_2_O for the synthesis of 3-D network-like mesoporous nanostructures. For the initial formation of binary metal hydroxides or metal carbonate hydroxides, reagents like NH_3_, urea, NaOH, NH_4_HCO_3_, NH_4_F, hexamethylenetetramine (HMTA) [103], diethylene glycol (DEG), cetyltrimethylammonium bromide (CTAB) [104], sodium dodecyl sulfate (SDS) [105], poly(diallyldimethylammonium chloride) (PDDA) [106], glycine [107], methyl glycerate [108], and ethylene glycol are added in the reaction mixture. The combination of some polar solvents such as ethanol, ethanol, propanol, ethylene glycol, and acetone along with water has also been found to facilitate the morphological characteristics [109]. Water:polar solvent ratio also significantly affects the growth mechanism. In Fig. 3a–d, different morphologies for the NiCo_2_O_4_ nanostructures are shown for water:ethanol ratios 1:0, 3:1, 1:1, and 1:3. More porous, denser, and thinner sheets were formed for the synthesized 3D flower-like NiCo_2_O_4_ nanostructures as the composition of ethanol was increased.

In the hydrothermal growth, the temperature is also a key factor in controlling the morphology of the nanostructures. Urchin- and sheaf-like NiCo_2_O_4_ nanostructures were synthesized by Umeshbabu et al. [104] using CTAB as a surfactant under hydrothermal conditions at 120  °C and 200  °C temperatures, respectively. Different morphologies were attributed to different degrees of crystal splitting and anisotropic crystal growth at different growth temperatures [110]. Further, the temperature also affects the magnitude of the van der Waals forces, hydrogen bonding, hydrophobic attraction, crystal field attraction, and intrinsic crystal contraction which subsequently control the Ostwald ripening process [111, 112].

Nayak et al. [89] mixed Ni(NO_3_)_2_·6H_2_O and Co(NO_3_)_2_·6H_2_O salts in a 1:2 atomic ratio along with urea which produced OH^−^ ions in the reaction mixture according to Eqs. 1–3.

1 $${\text{CO(NH}}_{ 2} )_{ 2} + {\text{H}}_{ 2} {\text{O}} \to 2 {\text{NH}}_{ 3} + {\text{CO}}_{ 2}$$

2 $${\text{NH}}_{ 3} {\text{ + H}}_{ 2} {\text{O }} \to {\text{NH}}_{ 4} {\text{OH}}$$

3 $${\text{NH}}_{ 4} {\text{OH }} \to {\text{NH}}_{ 4}^{ + } + {\text{OH}}^{ - }$$

Ni^2+^ and Co^2+^ on reaction with these OH^−^ ions formed Ni–Co bimetallic hydroxide [NiCo_2_(OH)_6_] which were finally converted into NiCo_2_O_4_ nanoneedles after crystal growth and calcinations. However, according to some reports, in the presence of urea, metal carbonate hydroxides are initially formed instead of bimetallic hydroxides (Eqs. 4–7) [113].

4 $${\text{CO}}_{ 2} { + } {\text{OH}}^{ - } \to {\text{HCO}}_{ 3}^{ - } \to {\text{H}}^{ + } { + } {\text{CO}}_{ 3}^{ 2- }$$

5 $$2 {\text{Ni}}^{2 + } + {\text{CO}}_{ 3}^{2 - } + 2 {\text{OH}}^{ - } \to {\text{Ni}}_{ 2} ( {\text{CO}}_{ 3} ) ( {\text{OH)}}_{ 2}$$

6 $$2 {\text{Co}}^{2 + } + {\text{CO}}_{ 3}^{2 - } + 2 {\text{OH}}^{ - } \to {\text{Co}}_{ 2} ( {\text{CO}}_{ 3} ) ( {\text{OH)}}_{ 2}$$

7 $${\text{Ni}}_{ 2} \left( {{\text{CO}}_{ 3} } \right)\left( {\text{OH}} \right)_{ 2} + 2 {\text{Co}}_{ 2} \left( {{\text{CO}}_{ 3} } \right)\left( {\text{OH}} \right)_{ 2} + {\text{O}}_{ 2} \to 2 {\text{NiCo}}_{ 2} {\text{O}}_{ 4} + 3 {\text{CO}}_{ 2} + 3 {\text{H}}_{ 2} {\text{O }}$$

**Fig. 4:**
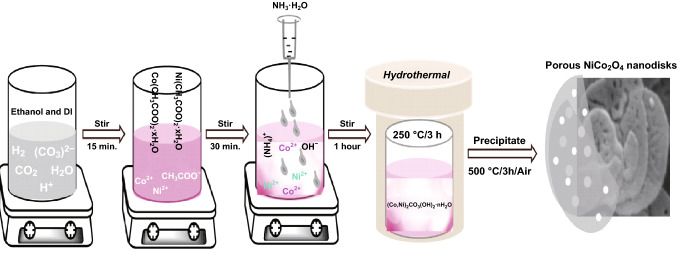
Schematic diagram for the synthesis of two-dimensional porous nanodisks of NiCo_2_O_4_. Reproduced with permission from Ref. [114], Copyright © 2018 Elsevier B.V.

Even ethanol as the solvent can also initiate the formation of metal carbonate hydroxides. Two-dimensional porous NiCo_2_O_4_ nanodisks were synthesized by a low-temperature hydrothermal method by Jain et al. [114] (Eqs. 8, 9). Figure 4 proposes the initial formation of Ni_2_(CO_3_)(OH)_2_ and Co_2_(CO_3_)(OH)_2_. Subsequent hydrothermal treatment in basic medium followed by calcination at 500 ^o^C formed two-dimensional porous NiCo_2_O_4_ nanodisks.

8 $${\text{C}}_{ 2} {\text{H}}_{ 5} {\text{OH}} + 3 {\text{H}}_{ 2} {\text{O}} \to 2 {\text{CO}}_{ 2} + 6 {\text{H}}_{ 2}$$

9 $${\text{CO}}_{ 2} + {\text{H}}_{ 2} {\text{O}} \to {\text{H}}_{ 2} {\text{CO}}_{ 3} \to 2 {\text{H}}^{ + } + {\text{CO}}_{ 3}^{2 - }$$

The nature of alkali source, capping agent, and other additives significantly affects the morphology of the NiCo_2_O_4_ nanostructures. Wang et al. [99] reported tremella-like NiCo_2_O_4_ nanostructures in the presence of HMTA, which transformed into flower-like nanostructures when NH_4_F was also added along with HMTA. However, when HMTA was replaced with urea, urchin-like and pine needle-like NiCo_2_O_4_ nanostructures were formed, respectively, in the absence and presence of NH_4_F additive [99]. HMTA is hydrolyzed to produce NH_3_ which finally produces OH^−^ ions as stated earlier in this section (Eq. 10).

10 $$( {\text{CH}}_{ 2} )_{ 6} {\text{N}}_{ 4} + 6 {\text{H}}_{ 2} {\text{O}} \to 4 {\text{NH}}_{ 3 } + 6 {\text{HCHO}}$$

It was suggested that the F^−^ ions released from NH_4_F stimulate the initially formed nanosheets and nanoneedles to produce more active sites to further activate nucleation, more mass loading of active material per unit area, firm binding between the active material, and hence more crystal growth [115–117]. The possible set of reactions elaborating the role of F^−^ ions released from NH_4_F is shown as follows [118] (Eqs. 11–13).

11 $${\text{Co}}^{2 + } + {\text{Ni}}^{2 + } + 2 {\text{F}}^{ - } \to {\text{CoF}}^{ + } + {\text{NiF}}^{ + }$$

12 $${\text{CoF}}^{ + } + {\text{NiF}}^{ + } + {\text{OH}}^{ - } \to {\text{CoF}}\left( {\text{OH}} \right) + {\text{NiF}}({\text{OH}})$$

13 $$4{\text{CoF}}\left( {\text{OH}} \right) + 2{\text{NiF}}\left( {\text{OH}} \right) + {\text{O}}_{2} \mathop{\longrightarrow}\limits^{\text{Annealing}}2{\text{NiCo}}_{2} {\text{O}}_{4 } + 6{\text{HF}}$$

**Fig. 5:**
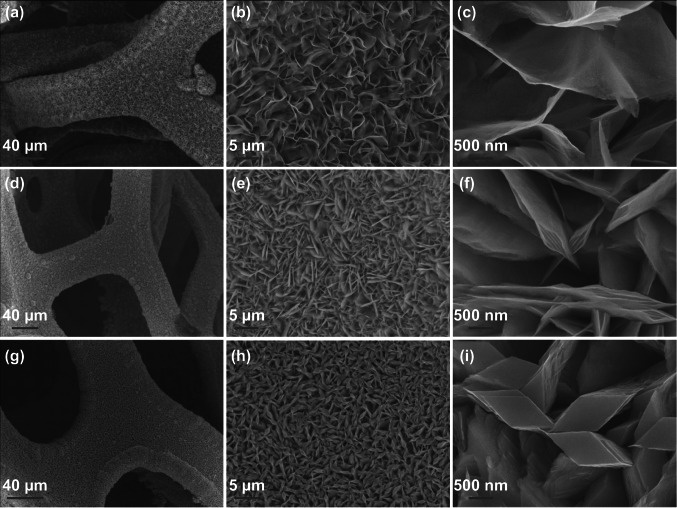
FESEM images representing the effect of concentration of NH_4_F on the morphologies of NiCo_2_O_4_ nanostructures: **a–c** 3 mmol NH_4_F; **d–f** 9 mmol NH_4_F; **g–i** 12 mmol NH_4_F. Reproduced with permission from Ref. [117]. Copyright © 2014 Elsevier Ltd.

Further, different concentrations of the NH_4_F also stimulated the initially formed nanostructures to acquire more versatile morphologies. For 3, 9, and 12 mmol concentrations of NH_4_F, various morphologies of the NiCo_2_O_4_ nanostructures are shown in Fig. 5. With an increase in concentration from 3 to 9 mmol, aggregation of the neighboring nanosheets occured. Further increase in concentration to 12 mmol, rhombus-shaped architectures were formed [117].

**Fig. 6:**
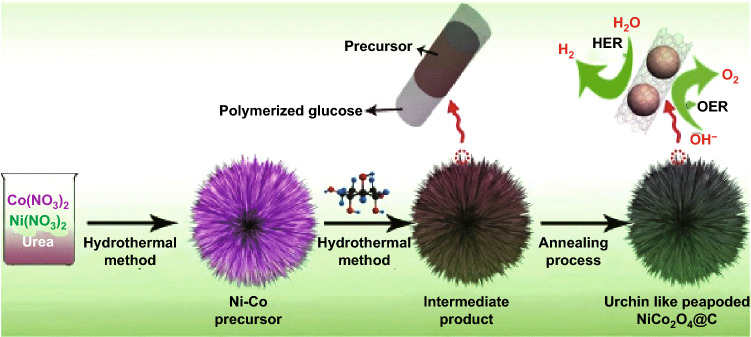
Schematic diagram of the process of urchin-like peapoded NiCo_2_O_4_@C. Reproduced with permission from Ref. [119], Copyright © 2017 Elsevier B.V.

Deng et al. [119] prepared novel urchin-like peapoded NiCo_2_O_4_@C nanostructures as a bifunctional catalyst for the water-splitting reaction. A three-phase process was proposed which included the initial hydrothermal synthesis of nanoneedles self-assembled microsphere followed by coating with polymerized glucose as green carbon source onto NiCo_2_O_4_ microsphere. The final stage was the calcination of the coated NiCo_2_O_4_ microsphere under N_2_ atmosphere to give urchin-like peapoded NiCo_2_O_4_@C. The fabrication process of urchin-like peapoded NiCo_2_O_4_@C is pictorially demonstrated in Fig. 6.

**Fig. 7:**
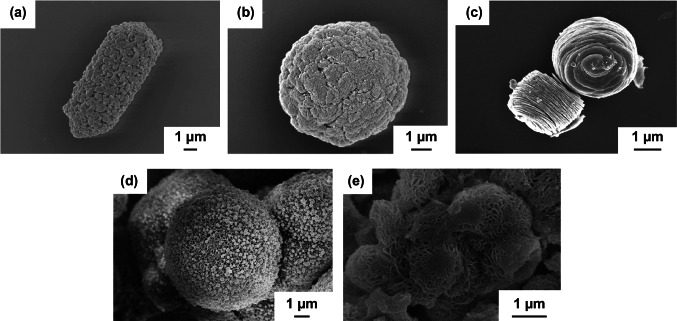
FESEM images of NiCo_2_O_4_ architectures prepared solvothermally using different solvents **a** water, **b** isopropanol, **c** 1:1 ethanol: water, **d** pure ethanol, and **e** diethylene glycol. Reproduced with permission from Ref. [122]. Copyright © 2017 American Chemical Society

Still another way of engineering the morphology, porosity, and growth of the crystals along the particular oriented crystal planes of the nanomaterials, is the use of non-aqueous solvents. The modified method is named as solvothermal instead of hydrothermal. Solvents with different solubilities and polarities can significantly affect the degree of supersaturation, the diffusion rates of the chemical species to the surface of the growing crystals, the interfacial surface energy, etc. [120, 121]. Fu et al. [122] synthesized 1D porous NiCo_2_O_4_ microrods (using metal acetate salts) (Fig. 7a) and microspheres (using metal nitrate salts) (Fig. 7b) in aqueous and isopropanol media, respectively, under similar conditions of temperature and reaction time. In 1:1 ethanol:water medium, spindle-like hierarchical architectures composed of closely packed microplates aligned along one direction with sizes of 3–5 μm were formed (Fig. 7c). In pure ethanol microspheres composed of nanosheets, interweave together with an average diameter of 8 μm were formed (Fig. 7d). However, in diethylene glycol, irregular aggregates with sheet-like structures were synthesized (Fig. 7e).

**Fig. 8:**
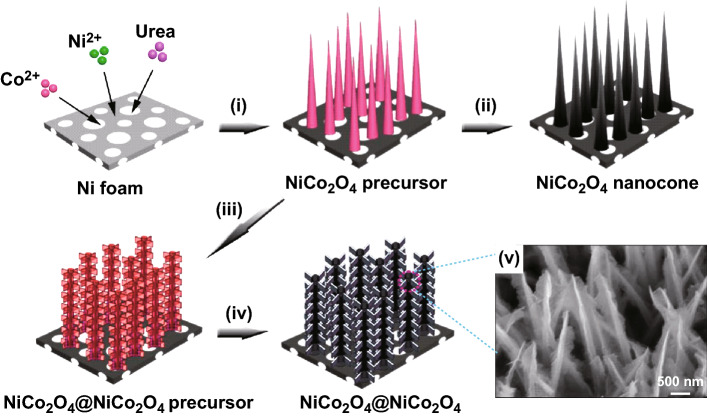
Schematic illustration for the formation of highly ordered 3D hierarchical NiCo_2_O_4_@NiCo_2_O_4_ core–shell nanocones arrays on nickel foams. Reproduced with permission from Ref. [123], Copyright © 2018 Elsevier B.V.

Wang et al. [123] in an interesting stepwise hydrothermal growth synthesized layers of NiCo_2_O_4_ nanosheets on the surface of NiCo_2_O_4_ nanocones precursor to give highly ordered 3D hierarchical NiCo_2_O_4_@NiCo_2_O_4_ core–shell nanocone arrays on nickel foams (Fig. 8). Different morphologies were engineered by controlling the reaction time and the temperature during stepwise hydrothermal growth. Further, NiCo_2_O_4_ nanocones arrays on Ni foam were synthesized in the absence of HMTA while the NiCo_2_O_4_ nanosheets growth on NiCo_2_O_4_ nanocones was guided by the presence of HMTA.

### Templated Solution Growth Method

**Fig. 9:**
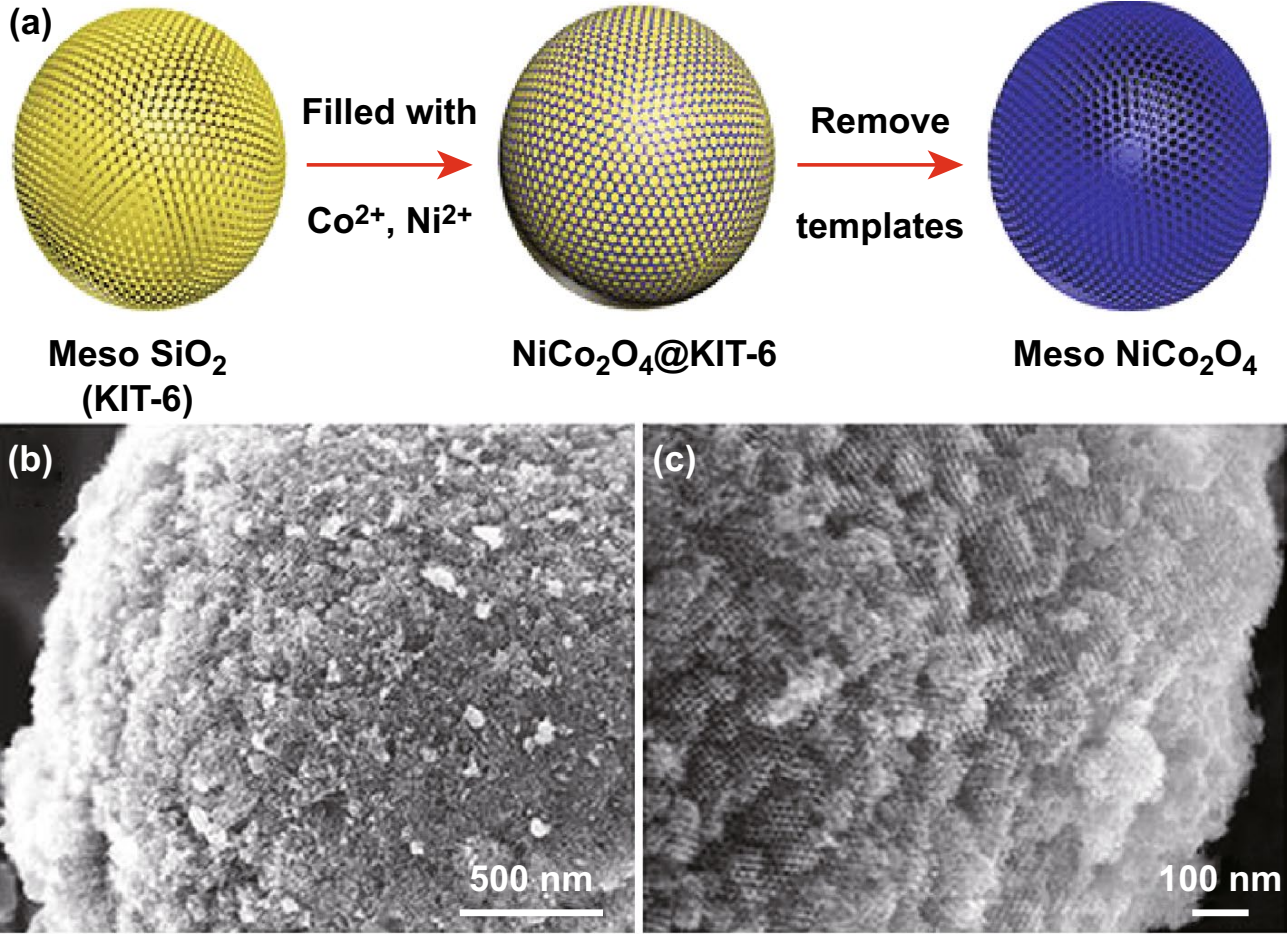
**a** Schematic illustration of the formation of mesoporous NiCo_2_O_4_ microspheres, **b** high-magnification FESEM image, and **c** TEM image of the mesoporous NiCo_2_O_4_ microspheres. Reproduced with permission from Ref. [125]. Copyright © Authors

The morphology, size, shape, and surface area of nanostructures can be designed through template-based synthesis to produce nanostructures with controlled physical, chemical, electrical, and electronic properties essential in notable applications and are also quite different from those of the bulk materials. Generally, three stages, viz., template preparation, directed synthesis of the desired material using the template, and the template removal, are described in the overall growth process of nanostructures [124]. The chemical nature, structure, concentration, and growth temperature are some of the important environmental factors affecting the growth of nanomaterials. Template-based methodologies are reported in the literature which govern the synthesis of NiCo_2_O_4_ nanomaterials with versatile morphologies including nanospheres, hollow spheres, nanocages, hollow submicron spheres, hollow irregular octahedra-like cages, flower-like nanostructure, microspheres with highly ordered mesoporous structures, nanowires, etc. With the development of new methods for synthesizing mesoporous binary NiCo_2_O_4_ metal oxides, the combination of template method with other methods such as hydrothermal/solvothermal, sol–gel has been widely used. In one such study, Ren et al. [125] prepared mesoporous NiCo_2_O_4_ microspheres using a mesoporous silica (KIT-6) template. The KIT-6 template was added into the metal nitrate precursor solution prepared in ethanol. The schematic illustration of the formation of mesoporous NiCo_2_O_4_ microspheres is shown in Fig. 9a. The high porosity of the synthesized mesospheres was ascertained by FESEM and TEM images (Fig. 9b, c). The template was finally removed by etching with 2 M NaOH solution [125].

**Fig. 10:**
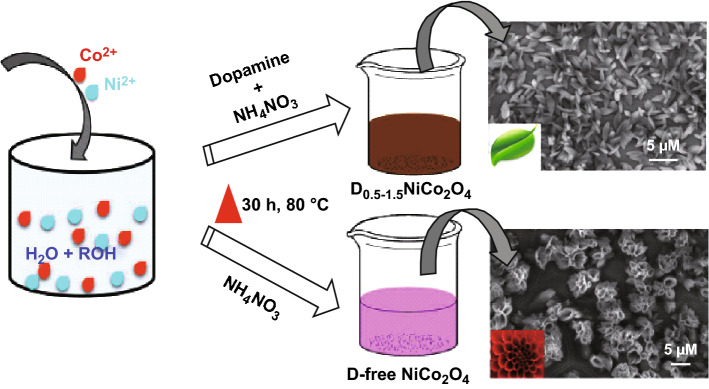
Schematic illustration of the synthesis of dopamine-free and dopamine-NiCo_2_O_4_ nanostructures. Reproduced with permission from Ref. [128], Copyright © 2016 American Chemical Society

Yuan et al. [126] utilized silica spheres as hard templates prepared by the modified Stöber method [127], for the synthesis of hierarchical mesoporous hollow NiCo_2_O_4_ submicron spheres with uniform size and mesoporous textual property. These submicron spheres were composed of ultrathin nanosheets with a thickness of a few nanometers. The NaOH solution was used for the in situ removal of silica spheres. Dopamine—a biomolecule containing amine functional groups is capable of self-polymerize under alkaline conditions. It forms a layer of the polydopamine which attracts various metal ions including Co^2+^ and Ni^2+^ cations due to strong electrostatic interactions. Further, the alkalinity of the medium results in the formation of –OH–Ni–OH–Co–OH– complex networks. This property has been explored for the synthesis of NiCo_2_O_4_ nanostructures by Veeramani et al. [128]. FESEM images shown in Fig. 10 are demonstrating the effect of dopamine on the morphology of the NiCo_2_O_4_ nanostructures. Flower-like dopamine derived NiCo_2_O_4_ nanostructures were formed.

**Fig. 11:**
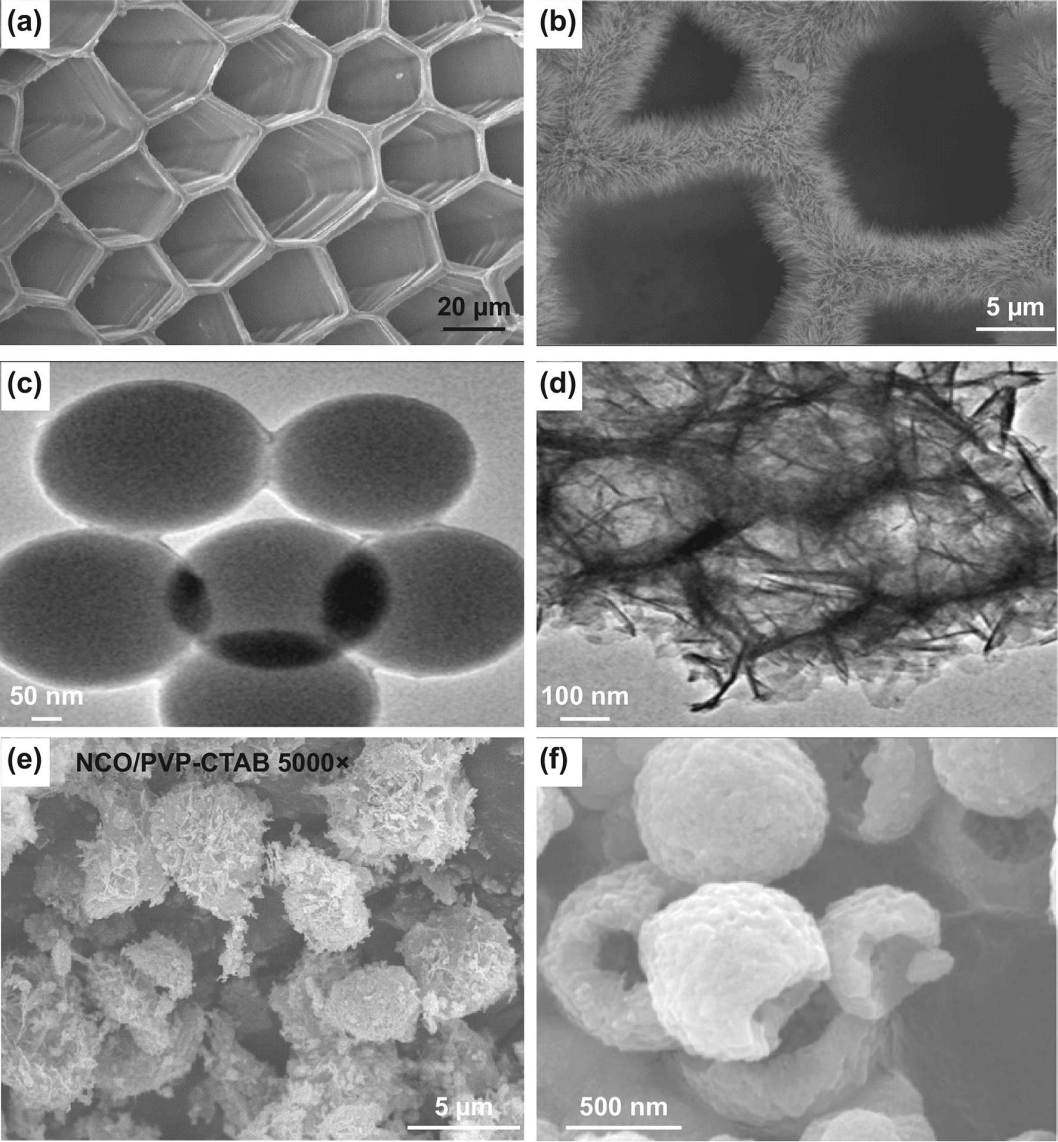
FESEM images of **a** MSBPC, **b** NiCo_2_O_4_ nanowires grown on MSBPC. Reproduced with permission from Ref. [129]. Copyright © 2014 American Chemical Society. **c** TEM image of SiO_2_@RF spheres, **d** TEM image of NiCo_2_O_4_ nanoflakes grown on SiO_2_@RF spheres. Reproduced with permission from Ref. [130]. Copyright © 2018 Elsevier B.V. **e** FESEM image of micron-sized NiCo_2_O_4_ pompon. Reproduced with permission from Ref. [132]. Copyright © 2019 Elsevier B.V. **f** FESEM image of NiCo_2_O_4_ hollow submicron spheres. Reproduced with permission from Ref. [133]. Copyright © 2015 Elsevier B.V.

In another significant strategy, Xiong et al. [129] used mollusk shell-based macroporous carbon material (MSBPC), as a template to grow NiCo_2_O_4_ nanowires hydrothermally (Fig. 11a, b). The MSBPC was obtained from mollusc shells by removing calcium carbonate crystal and other biomacromolecules by acid treatment and carbonization. It was observed that there was uniform and dense growth of the NiCo_2_O_4_ nanowires on the inner walls of MSBPC channels. The average length of the NiCo_2_O_4_ nanowires was about 1.5 µm. Li et al. [130] reported the synthesis of composite C@NiCo_2_O_4_ hollow microspheres via a two-step strategy of hard template-induced hydrothermal synthesis followed by calcination. SiO_2_@RF (resorcinol–formaldehyde resin, RF) sphere was used as a hard template, whereas HMTA was used as precipitant. The template SiO_2_@RF was synthesized via a one-pot sol–gel process under alkaline condition using an alcohol–water mixed solvent [131]. The SiO_2_ core was removed by treating the prepared material with 2 M NaOH at room temperature for 12 h. The SiO_2_@RF template was having a core–shell structure with an average diameter of 350 nm (Fig. 11c). The NiCo_2_O_4_ nanoflakes were grown and assembled on the carbon surface of the SiO_2_@RF spheres (Fig. 11d). Recently, novel micron-sized NiCo_2_O_4_ pompon was prepared by templated growth using polyvinylpyrrolidone (PVP) non-ionic polymer and cationic surfactant CTAB as co-template [132]. Columbic and coordinative forces between template, co-template, and the metal ions help to form a stable “hairball” structure which finally was converted into a micron-sized pompon-like product on annealing (Fig. 11e). In contrast, in the absence of co-template CTAB, mesoporous NiCo_2_O_4_ hollow submicron spheres with a uniform diameter of 400–500 nm were obtained through a soft template method assisted by PVP (Fig. 11f). Further, in the absence of even PVP, solid submicron spheres were obtained [133].

**Fig. 12:**
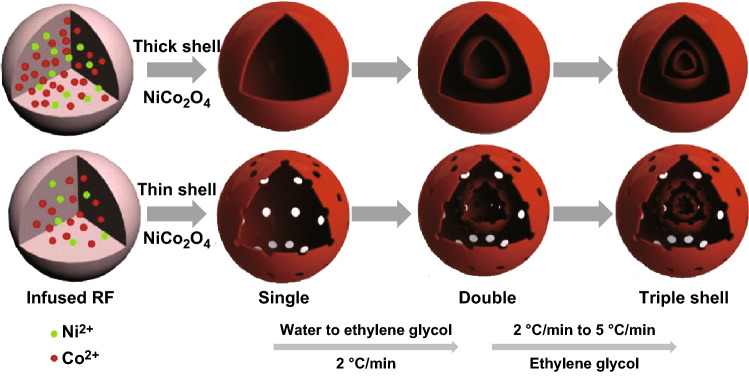
Pictorial representation for the formation of NiCo_2_O_4_ hollow microspheres with tunable numbers and shell thickness. Reproduced with permission from Ref. [131]. Copyright © 2016 Elsevier B.V.

Qi et al. [131] also used RF microspheres as templates for the synthesis of NiCo_2_O_4_ hollow microspheres with tunable shell numbers and shell thickness. The shell numbers were controlled by adjusting the solvent ratio (DI water: ethylene glycol) and heating ramp rate, whereas the shell thickness and porosity were controlled by adjusting the metal ion concentrations (Fig. 12). For total molar concentrations of Ni^2+^ and Co^2+^ of 0.05 and 0.1 M, thin and thick shells, respectively, were formed. NiCo_2_O_4_ hollow microspheres with double and triple shells were formed at a heating ramp rate of 2 and 5 °C min^−1^, respectively, in EG as a solvent. With the increase in the ramp rate, the increased temperature gradient of the infused RF microspheres along the radial direction favors the separation of adjacent NiCo_2_O_4_ layers and the infused RF cores, thereby transforming double shell to triple shells [134]. Furthermore, EG prevents the formation of the metal aqua ions, and thus, the penetration of the metal ions into RF microspheres is accelerated which is essential for the formation of multi-shell NiCo_2_O_4_ hollow microspheres [135, 136]. Additionally, the final calcination process also results in some adhesion force in the outward direction and the contraction force by decomposition of the inner core which segregates the outer NiCo_2_O_4_ shell and the inner infused RF [131].

In addition to templates of organic origin, inorganic metal oxides have also been reported as template materials for the synthesis of NiCo_2_O_4_ nano-/microarchitectures [137]. Lv et al. [138] synthesized hollow NiCo_2_O_4_ octahedral nanocages via a Cu_2_O-templated strategy in combination with a coordination reaction. Uniform Cu_2_O octahedral crystals were prepared by reducing a copper-citrate complex solution with ascorbic acid in the presence of PVP. Initially, amorphous (NiCox)O(OH) was deposited onto the Cu_2_O octahedral crystals through a precipitation method. Cu_2_O octahedral crystals were etched according to a “coordinating etching and precipitating” (CEP) using Na_2_S_2_O_3_ as coordinating etchant [139] (Eqs. 14, 15). After that, the product was annealed at 400 °C for 2 h to get the hollow NiCo_2_O_4_ nanocages. In a similar study, Huang et al. [140] reported the synthesis of highly porous NiCo_2_O_4_ hollow nanospheres through a polycrystalline Cu_2_O-templated route based on “coordinating etching and precipitating” process. The excellent electron transfer capability, large specific surface area, and intrinsic redox couples of Ni^2+^/Ni^3+^ and Co^2+^/Co^3+^ ions, and superior electrocatalytic activity of NiCo_2_O_4_ hollow nanospheres were explored for glucose sensing by cyclic voltammetry and electrochemical impedance spectroscopy. NiCo_2_O_4_ hollow nanosphere-modified glassy carbon electrode (GCE) exhibited a high sensitivity of 1917 μA mM^−1^ cm^−2^, linear dynamic ranges of 0.01–0.30 mM and 0.30–2.24 mM, and very low detection limit of 0.6 μM (S/N = 3). Solid CuO octahedral is also reported as template materials for the synthesis of hollow octahedra-like NiCo_2_O_4_ cages. However, CuO templates can be simply removed by dissolving with a diluted NH_4_OH solution [141].

14 $${\text{Cu}}_{2} {\text{O}} + {\text{S}}_{2} {\text{O}}_{3}^{2 - } + {\text{H}}_{2} {\text{O}} \to {\text{Cu}}_{2} ({\text{S}}_{2} {\text{O}}_{3} ) + 2{\text{OH}}^{ - }$$

15 $${\text{S}}_{2} {\text{O}}_{3}^{2 - } + {\text{H}}_{2} {\text{O}} \leftrightarrow {\text{HS}}_{2} {\text{O}}_{3}^{ - } + {\text{OH}}^{ - }$$

Yang et al. [142] reported NiCo_2_O_4_ hollow nanorods prepared by the sacrificial template-accelerated acid hydrolysis of ZnO (Eq. 16).

16 $${\text{ZnO}} + 2{\text{H}}^{ + } \to {\text{Zn}}^{2 + } + {\text{H}}_{2} {\text{O}}.$$

### Sol–gel Method

The sol–gel process represents the chemical conversion of the liquid “sol” to the network “gel” phase, subsequently post-treatment into solid metal oxides with microcrystalline ultrafine particles. It is superior to other methods because it can better control the texture and surface properties of synthesized nanomaterials. The sol–gel method for the synthesis of nanomaterials is affected by numerous factors including pH, temperature, nature of solvent, growth time, agitations time, presence of capping agents, template, etc. With the consideration of these factors and potential applications, many protocols have been used to design materials of different sizes and features, including nano-, micro-, meso-, and macro-materials. To get excellent porosity and conductivity for potential electrochemical applications, the addition of polymers stuffs such as PVP [143], organic solvents/additives like propionic acid [144], citric acid [145, 146], *N*,*N*-dimethylformamide (DMF) [147], and epoxides like propylene oxide [148, 149], during the post-annealing process is suggested. Significantly the additive/metal ion molar ratio is very important in controlling the pore size and pore volume. Traditional use of SiO_2_ is avoided as its addition decreases the conductivity and limits the connection of the film with conducting substrate in thin film forms of NiCo_2_O_4_ [143]. In a typical sol–gel method, the NiCo_2_O_4_ spinel oxide was prepared by mixing appropriate amounts of metal salt precursors along with citric acid. The resulting solution was magnetically stirred at 80  °C for 2 h to get a gelatinous matrix. Finally, the matrix was calcined at 550  °C for 5 h to get the desired product [146]. Citric acid was also used as a chelating ligand for the synthesis of highly porous coral-like crystalline NiCo_2_O_4_ nanoparticles with submicron sizes via a facile sol–gel method in H_2_O-DMF mixture as solvent [147]. Liu et al. prepared nanoporous NiCo_2_O_4_ thin films deposited on ITO glass. The precursor solutions for NiCo_2_O_4_ nanospheres were prepared via a sol–gel method in glacial acetic acid and ethanol as solvents, and ethylene glycol and CTAB were used as a viscosity modifier template, respectively [150]. Thus, the sol–gel process is a proven and important method for preparing NiCo_2_O_4_ nanoparticles.

### Co-precipitation Method

**Fig. 13:**
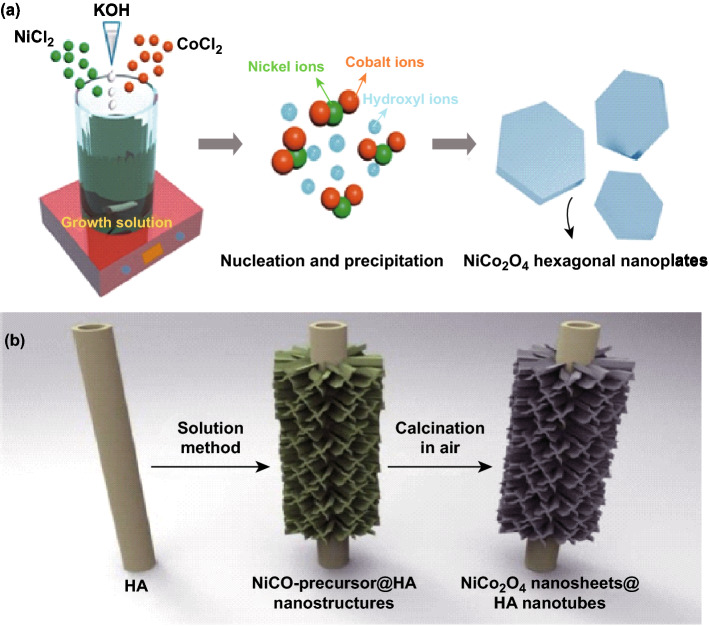
**a** Schematic representation for the synthesis of hexagonal NiCo_2_O_4_ nanosheets, Reproduced with permission from Ref. [151]. Copyright © 2019 Elsevier Ltd. and **b** hierarchical NiCo_2_O_4_ nanosheets@halloysite nanotubes via co-precipitation method. Reproduced with permission from Ref. [152]. Copyright © 2014 American Chemical Society

Better stoichiometric control and high purity of the metal oxide nanomaterials can be easily achieved through the coprecipitation method which involves simultaneous precipitation from a homogeneous solution of two or more cations. Simultaneous occurrence of nucleation, growth, coarsening, Ostwald ripening, and aggregation dramatically affect the size, morphology, and properties of the metal oxide nanoparticles. The technique has been applied for the synthesis of NiCo_2_O_4_ nanomaterials. NiCo_2_O_4_ hexagonal nanostructures were prepared by Bhagwan et al. [151] using Ni and Co chlorides and 6 M KOH as the precipitating agent. The schematic illustration for the formation of NiCo_2_O_4_ hexagonal is shown in Fig. 13a. It was suggested that the strong alkaline environment in the growth solution caused nickel and cobalt ions to precipitate and nucleate together, forming nickel–cobalt hydroxide which was subsequently converted into NiCo_2_O_4_ hexagonal after calcination at 300  °C. Liang et al. [152] reported hierarchical NiCo_2_O_4_ nanosheets@halloysite nanotubes (Fig. 13b). The initial formation of NiCo precursor@halloysite nanotubes was assisted by HMTA and dehydrated citric acid trisodium salt.

**Fig. 14:**
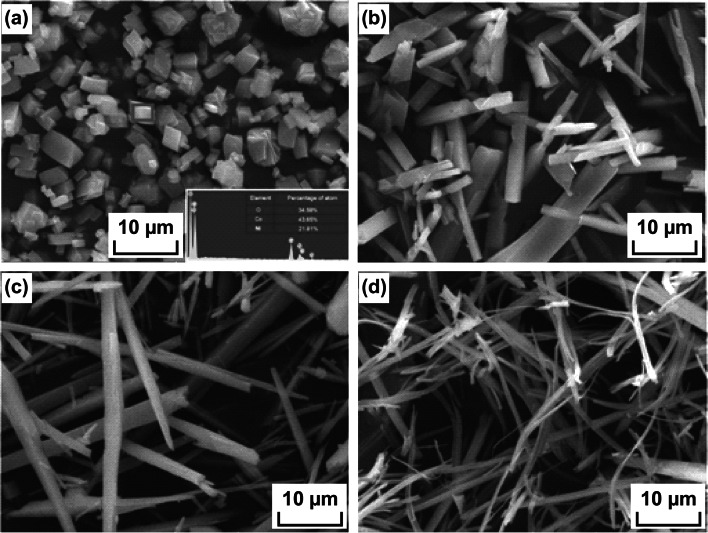
FESEM images NiCo_2_O_4_ precursor powders prepared at **a** pH = 3, **b** pH = 7, **c** pH = 8 and **d** pH = 8.4. Reproduced with permission from Ref. [159]. Copyright © 2018 Elsevier Ltd.

A stepwise co-precipitation template free method was designed by Chen et al. [153] for the synthesis of hierarchical urchin-like NiCo_2_O_4_ hollow nanospheres. Urea-assisted mesoporous urchin-like NiCo_2_O_4_ nanostructures were prepared by Jadhav et al. [154] by an easy, viable, and cost-effective co-precipitation method. Yu et al. [155] explored the structure-stabilizing properties of PVP, which can bind the metal ions through electrostatic interaction with the –N and/or C=O functional groups, for the formation of Ni–Co precursor particles with tetragonal prism-like shapes by a modified coprecipitation method. The yolk–shell Ni–Co oxide nanoprisms with a highly porous interior core structure consisting of numerous polycrystalline primary particles were obtained finally after annealing. Other stabilizing and precipitating agents like ethylene glycol (EG) [156], urea [157], NaOH, NH_4_OH, NH_4_HCO_3_, H_2_C_2_O_4_ [158, 159] and NaHCO_3_ [153] are reported in the literature. Organic stabilizers such as EG are supposed to form a protective layer around the particle surface through interactions with hydroxyl groups preventing the aggregation. Moreover, EG also acts as a bidentate chelating ligand for solvated metal ions [160]. Another important factor that controls the morphology, shape, and size of the nanoparticles is the pH of the reaction medium during coprecipitation. Wan et al. [159] observed the change in morphology of the NiCo_2_O_4_ precursors from the cubic to the fibrous along the axial direction. The fibrous morphology was maintained at a still higher pH value of 8.4; however, the aspect ratio was increased (Fig. 14a–d). A dynamic equilibrium was suggested to exist between metal ammoniated complexes and the coprecipitation of Ni^2+^ and Co^2+^ as their oxalates.

**Fig. 15:**
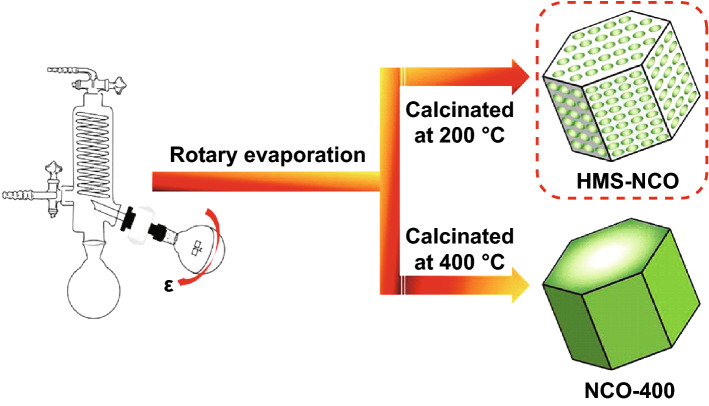
Schematic illustration of the synthesis of hexagonal mesoporous structured NiCo_2_O_4_ (HMS-NCO) and NiCo_2_O_4_ calcined at 400 °C (NCO-400). Reproduced with permission from Ref. [161]. Copyright © 2018 Elsevier Ltd and Techna Group S.r.l.

The post-annealing temperature is also an important factor for controlling the morphology of the NiCo_2_O_4_ spinel structures. The homogeneous dark blue-colored suspension which was obtained by mixing the metal nitrates and NaOH solution was initially evaporated under rotation and reduced pressure conditions by a cost-effective rotary evaporation method. Hexagonal column-like mesoporous loose architectures and hexagonal dense blocks were obtained at 200 and 400  °C calcination temperatures, respectively (Fig. 15) [161].

### Electro-Deposition

**Fig. 16:**
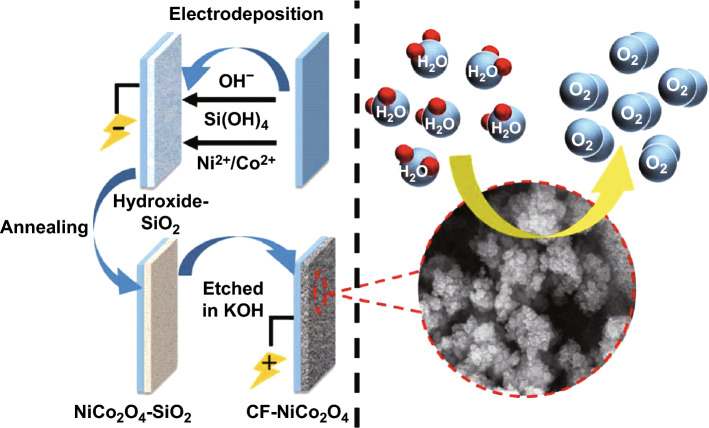
Schematic illustration of the formation of cauliflower-like NiCo_2_O_4_ film. Reproduced with permission from Ref. [165]. Copyright © 2017 Hydrogen Energy Publications LLC. Published by Elsevier Ltd

Electro-deposition is considered a very useful, versatile, and flexible tool for the deposition of dendritic hierarchical structures, thin and thick films, nanosheet, nanofoil, nanotubes, nanowires, and many well-ordered transition metal oxides on conducting surfaces. Potentiostatic, galvanostatic, and pulse plating are the three main techniques employed for electro-deposition [162, 163]. The basic principle of electro-deposition involves three steps, viz. preparation of a metal ions precursor solution, co-electro-deposition, and final thermal decomposition [164]. Recently, this technique has also been used for the preparation of NiCo_2_O_4_ spinel structures for various applications, including supercapacitors, anode materials for Li-ion batteries, gas sensors, biosensors, etc. Wu et al. [165] deposited nanostructured cauliflower-like NiCo_2_O_4_ film through galvanostatic electro-deposition combined with annealing treatment (Fig. 16). Galvanostatic electro-deposition was performed using a three-electrode compartment comprising a stainless steel disk as a working electrode. An Ag/AgCl saturated with KCl and a platinum plate were used as the reference and counter electrodes, respectively. Hydroxide-SiO_2_ template transformed nanoflakes to cauliflower-like NiCo_2_O_4_ nanoparticles. Under cathodic potential, the generated OH^−^ ions catalyzed the sol–gel process for the formation of SiO_2_. The generated OH^−^ ions facilitated the formation of Ni(OH)_2_ and Co(OH)_2_. Heat treatment of the deposited at 250 °C in air for 2 h converts the metal hydroxides into NiCo_2_O_4_ films.

Wang et al. [166] reported the electro-deposition of the nickel/cobalt/zinc ternary alloy layer on ultrafine nickel wire. Removal of the zinc by dealloying with NaOH solution followed by oxidation at the atmospheric environment resulted in mesoporous NiCo_2_O_4_ film on the surface of ultrafine nickel wire. Zhao et al. [167] grew NiCo_2_O_4_ nanosheet networks on carbon cloth through a simple cathodic electro-deposition process followed by post-annealing at 300  °C in an air atmosphere for 120 min. The average mass loadings for NiCo_2_O_4_ nanosheet networks grown on carbon cloth at different electro-deposition times 200, 400, and 600 s were 0.4, 0.6, and 0.9 mg cm^−2^, respectively. The ${\text{NO}}_{ 3}^{ - }$ ions from the metal salts were reduced to ${\text{NO}}_{ 2}^{ - }$ and ${\text{NH}}_{ 4}^{ + }$ ions at the cathode. This reduction also resulted in the formation of OH^−^ ions which combined with the Ni^2+^ and Co^2+^ to form amorphous binary metal hydroxide NiCo_2_(OH)_6_ nanosheet networks [168]. Post-annealing transforms the NiCo_2_(OH)_6_ into NiCo_2_O_4_ nanosheet networks [57, 169] (Eqs. 17–20).

17 $${\text{NO}}_{3}^{ - } + {\text{H}}_{2} {\text{O}} + 2{\text{e}}^{ - } \to {\text{NO}}_{2}^{ - } + 2{\text{OH}}^{ - }$$

18 $${\text{NO}}_{2}^{ - } + 6{\text{H}}_{2} {\text{O}} + 6{\text{e}}^{ - } \to {\text{NH}}_{4}^{ + } + 8{\text{OH}}^{ - }$$

19 $${\text{Ni}}^{2 + } + 2{\text{Co}}^{2 + } + 6{\text{OH}}^{ - } \to {\text{NiCo}}_{2} ({\text{OH}})_{6}$$

20 $${\text{NiCo}}_{2} ({\text{OH}})_{6} + \frac{1}{2}{\text{O}}_{2} \to {\text{NiCo}}_{2} {\text{O}}_{4} + 3{\text{H}}_{2} {\text{O}}$$

**Fig. 17:**
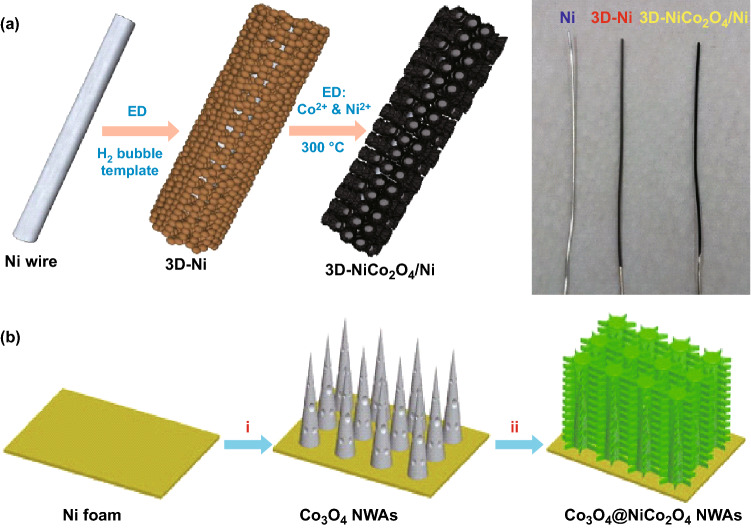
**a** Electrodeposited 3D flower-like NiCo_2_O_4_/Ni nanostructures on Ni-wire. Reproduced with permission from Ref. [169], Copyright © 2016 The Royal Society of Chemistry. **b** Schematic representation of the formation of nanoforest hierarchical composites Co_3_O_4_@NiCo_2_O_4_ nanowire arrays. Reproduced with permission from Ref. [172]. Copyright © 2013 Elsevier Ltd.

The dissolution of the ions decreases near the electrode due to the formation of the OH^−^ ions and an increase in pH near the electrode is observed. Since the solubility constants of Ni(OH)_2_ (8.2 × 10^−16^) and Co(OH)_2/3_ (2.5 × 10^−16^) are very low and comparable, their simultaneous precipitations occur which finally gives NiCO_2_(OH)_6_ [170, 171]. Ramadoss et al. [169] electrodeposited highly porous and binder-free 3D flower-like NiCo_2_O_4_/Ni nanostructures on Ni wire and explored their supercapacitor applications (Fig. 17a). The high porosity of the nanostructures was attributed to the presence of H_2_ bubbles produced by hydrogen evolution reaction during electro-deposition. Furthermore, H_2_ bubbles also acted as a template for the construction of a 3D flower-like NiCo_2_O_4_/Ni with dendritic walls on the Ni wire. Nanoforest hierarchical composites Co_3_O_4_@NiCo_2_O_4_ nanowire arrays were synthesized by Zhang et al. [172]. Co_3_O_4_ nanowires were initially grown on Ni foam through a facile hydrothermal method. After that, NiCo_2_O_4_ was electrochemically deposited in the Co_3_O_4_ nanowires to avoid the conventional aggregation (Fig. 17b). Mirzaee et al. [173] proposed a two-step method involving initial electro-deposition followed by thermal treatment at 300 °C with a ramping rate of 1 °C min^−1^ to form flower-like arrays of NiCo_2_O_4_ on electrochemically reduced graphene oxide (ERGO) which itself was deposited on nickel–nickel oxide foam.

In addition to these, NiCo_2_O_4_ architectures of versatile morphologies have been electrochemically deposited on a variety of conducting surfaces. Some of these include honeycomb-shaped NiCo_2_O_4_ on carbon cloth [174], ultrathin NiCo_2_O_4_ nanosheets on three-dimensional interwoven nitrogen-doped carbon nanotubes [175], ultrathin porous NiCo_2_O_4_ nanosheet arrays on flexible carbon fabric, 3D vertically aligned carbon nanotubes/NiCo_2_O_4_ core/shell structures [176], hybrid composite Ni(OH)_2_@NiCo_2_O_4_ on carbon fiber paper [177], 3D hierarchical NiCo_2_O_4_@MnO_2_ hybrid nanomaterial on stainless steel mesh [178], freestanding bowl-like NiCo_2_O_4_ on carbon fiber paper [179], network-like holey NiCo_2_O_4_ nanosheet arrays on Ni foam [180], NiCo_2_O_4_@ carbon nanofibers [181], and many more.

### Combustion Method

Combustion synthesis, also referred to as self-propagating high-temperature synthesis is one of the most versatile, convinced, convenient, cost-effective, and fast method for the synthesis of nanomaterials. It involves a thermally induced redox reaction between precursor salt as oxidizers and an organic fuel [182–184]. Glucose, fructose, tartaric acid, sucrose, glycine, citric acid, hydrazine, urea, and oxalic acid are generally used as organic fuels. However, if metal oxalate or acetate salts are used, the combustion process can be directly conducted in the absence of fuel [185]. Byproduct gases like CO_2_, H_2_O, N_2_, oxides of N (NO_x_) and S (SO_x_), etc. are evolved during the combustion process [186]. The release of these gases promotes the expansion of the product and rapid fall in temperature after the reaction ceases. This provides a solid product with a high degree of porosity and good dispersibility [187]. As compared to solid-state combustion, liquid phase combustion synthesis has proved to be the most suitable one as oxidizers and fuel are well dissolved in aqueous or alcoholic solutions [188]. Ni(NO_3_)_2_·6H_2_O, Co(NO_3_)_2_·6H_2_O (in 1:2 molar ratio) as oxidizers and tartaric acid as fuel were dissolved in acidified 2-methoxy ethanol solution. The resulting solution was combusted at 250 °C for 1 h to prepare NiCo_2_O_4_ nanoparticles [189]. Sucrose assisted combustion of the Ni and Co nitrates also resulted in NiCo_2_O_4_ nanoparticles when the combustion process was carried out at 350 °C for 6 h [190]. The oxalate precursors were directly decomposed into NiCo_2_O_4_ powders by heating in an air ambient atmosphere at 320 °C for 10 h [185]. Citric acid assisted combustion at 400 °C for 4 h resulted in highly porous NiCo_2_O_4_ nanomaterials [191]. Urea-assisted combustion was processed at 400 °C for 2 h in ethyl acetate as a solvent [192]. In each case, a viscous gel is obtained initially by heating the reaction solution at low temperature followed by auto-ignition resulting in the formation of highly fluffy mass which is finally calcined at high temperature. Direct calcination of the metal nitrate salts in the presence of alkalis without any fuel has also been reported for the synthesis of the NiCo_2_O_4_ nanorods [41].

Though it is a fast and low-cost method for the synthesis of NiCo_2_O_4_ powders, it suffers from some major drawbacks including less control over morphological uniformity and particle size, the simultaneous formation of a variety of crystal phases, the formation of highly agglomerated structures, complex and uncertain growth mechanism, and critically very low possibilities of formation of a versatile and wide range of morphological structures as those of in hydrothermal and other solution methods.

### Electro-Spinning Method

**Fig. 18:**
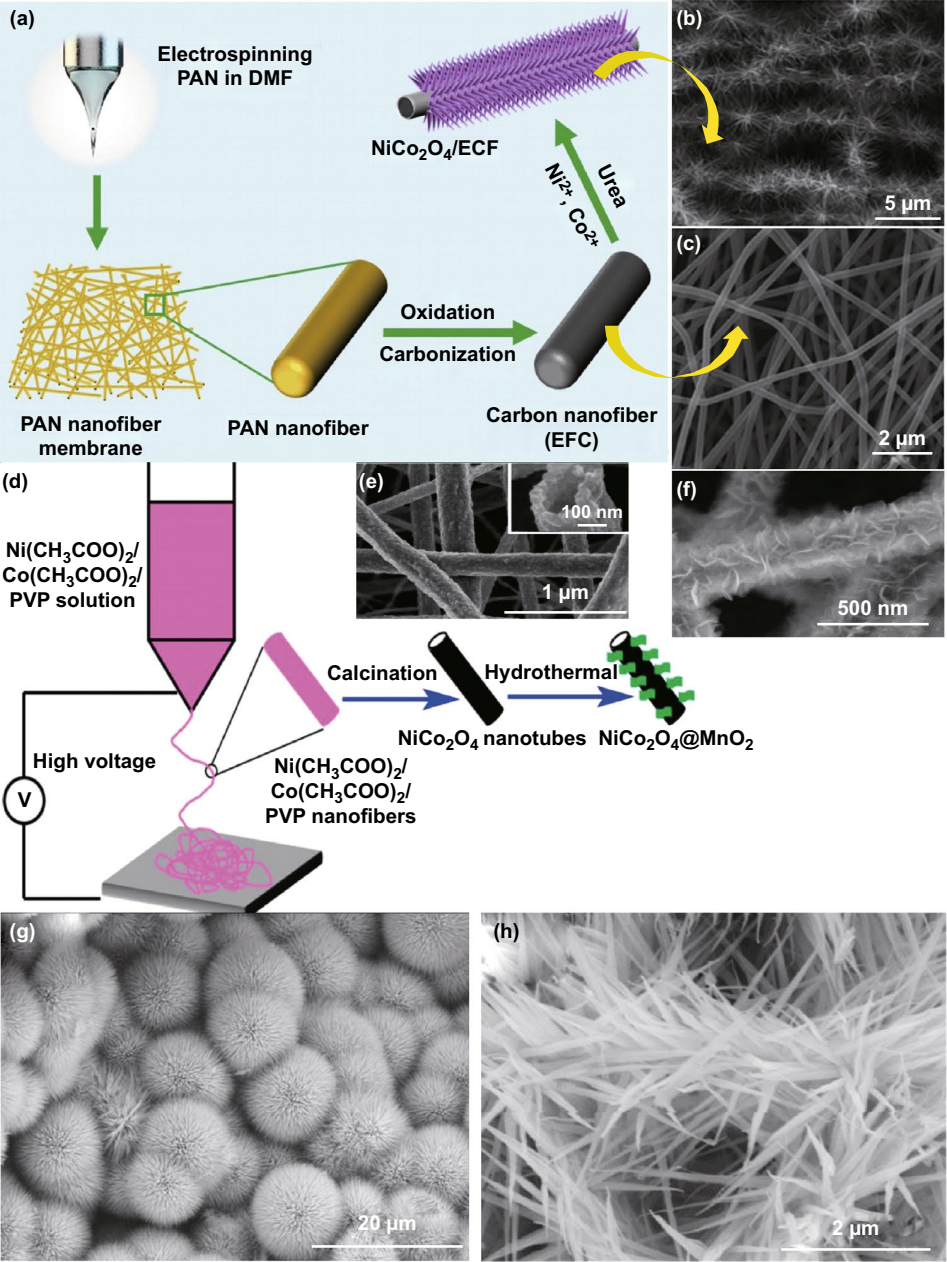
**a** Fabrication procedure of NiCo_2_O_4_/ECF nanohybrids, **b** FESEM image of ECF and **c** FESEM image of NiCo_2_O_4_ nanoneedles grown on ECF. Reproduced with permission from Ref. [39]. Copyright © 2017 Elsevier B.V. **d** The fabrication procedure for NiCo_2_O_4_@MnO_2_ composites, **e** FESEM image of NiCo_2_O_4_ nanotubes and **f** FESEM image of MnO_2_ nanosheets grown of NiCo_2_O_4_ nanotubes. Reproduced with permission from Ref. [200]. Copyright © 2016 Elsevier B.V. **g, h** Low- and high-magnification FESEM images, respectively, of NiCo_2_O_4_ with uniform dandelion-like morphologies. Reproduced with permission from Ref. [203]. Copyright © 2019 Elsevier Ltd.

Many electrospun carbonaceous materials such as carbon nanofibers, single-walled carbon nanotubes, multi-walled carbon nanotubes, etc. prepared from oxidation and carbonization of polymers like PVP, PAN, PVA have been used as templates for the growth and deposition of NiCo_2_O_4_ nanostructures with versatile morphologies. In one synthetic way, there is simultaneous growth of NiCo_2_O_4_ nanostructures and electro-spinning of template material [193, 194]. In another strategy, NiCo_2_O_4_ nanostructures are grown through other synthetic methods like hydrothermal, sol–gel, coprecipitation, etc. on pre-electrospun carbonaceous templates [39]. Electro-spinning setup comprises a high-voltage system, spinneret, and collector which results in the formation of continuous nanofibers with diameters ranging from nanometer to micrometer [195–197]. The deposition of NiCo_2_O_4_ nanostructures on these carbonaceous materials not only improves the electrical and electronic properties but also enhances the thermal, mechanical and chemical stabilities which are the important prerequisite characteristics for the biosensing and other applications. The composition of the precursor solution, presence of additives like templates and capping agents, modification in the electro-spinning setup, post-annealing, electrospun voltage are some of the major factors which control the thickness, porosity, and morphology of the deposited NiCo_2_O_4_ films. Lai et al. [198] through electro-spinning, co-deposition, redox deposition fabricated NiCo_2_O_4_-doped carbon nanofiber@MnO_2_ nanosheet and nanorod hybrid membranes. Busacca et al. [199] prepared NiCo_2_O_4_/carbon nanofibers composites and investigated their oxygen evolution reaction in alkaline electrolyte. Metal acetate salt precursor in a molar ratio 1:2 was mixed in PAN (as carbon source) and DMF. The electrospun layer was thermally oxidized at 270 °C in air for 30 min followed by subsequent carbonization at 900 °C for 1 h under a helium gas flow. Li et al. [193] fabricated porous one-dimensional NiCo_2_O_4_ nanostructures via a single-spinneret electro-spinning method. Stoichiometric amounts of Ni and Co nitrates were homogeneously mixed in a solution prepared by dissolving PVP in ethanol and *N*,*N*-dimethylformamide. Metallic precursor concentration: PVP (M: PVP) ratio was significant in determining the morphologies of the electrospun one-dimensional NiCo_2_O_4_ nanostructures. For 0.44:1, 0.61:1, and 0.87:1 M: PVP ratios, NiCo_2_O_4_ nanofibers, nanotubes, and nanobelts were formed. The versatility in morphologies was attributed to the fast water evaporation and burning off of PVP during annealing. Guan et al. [194] synthesized spinel NiCo_2_O_4_ nanofibers with diameters of 50–100 nm through electro-spinning of the PVA/cobalt acetate/nickel acetate composite precursor followed by annealing at high temperatures ranging from 400 to 800 °C. Liu et al. [39] demonstrated the surfactant-assisted hydrothermal uniform growth NiCo_2_O_4_ nanoneedle on electrospun carbon nanofiber (ECF) and explored their glucose sensing properties non-enzymatically. ECF film was prepared through initial electro-spinning and subsequent oxidation and carbonization of PAN (Fig. 18a–c). Xu et al. [200] instead of PAN used PVP as a carbon source to produce NiCo_2_O_4_ nanotubes. These nanotubes were used as scaffolds for hydrothermal growth of MnO_2_ nanosheets for the additional improvement in electronic conductivity and electrochemical activity for supercapacitor applications (Fig. 18d–f). Copolymers like poly (acrylonitrile-co-methylhydrogen itaconate) [201] and biobased polymer composites such as PAN/lignin [202] are also reported in the literature for the formation of flexible carbon nanofibers. The hollow carbon nanofibers were used as a template for the hydrothermal growth of NiCo_2_O_4_ with uniform dandelion-like morphology consisting of densely grown nanoneedle (Fig. 18g, h) [203]. The above discussion thus reveals that the proper combination and the composition of the polymers can result in the formation of carbonaceous materials with versatile structural features with high surface area necessary for potential applications.

### Microwave-Assisted Method

**Fig. 19:**
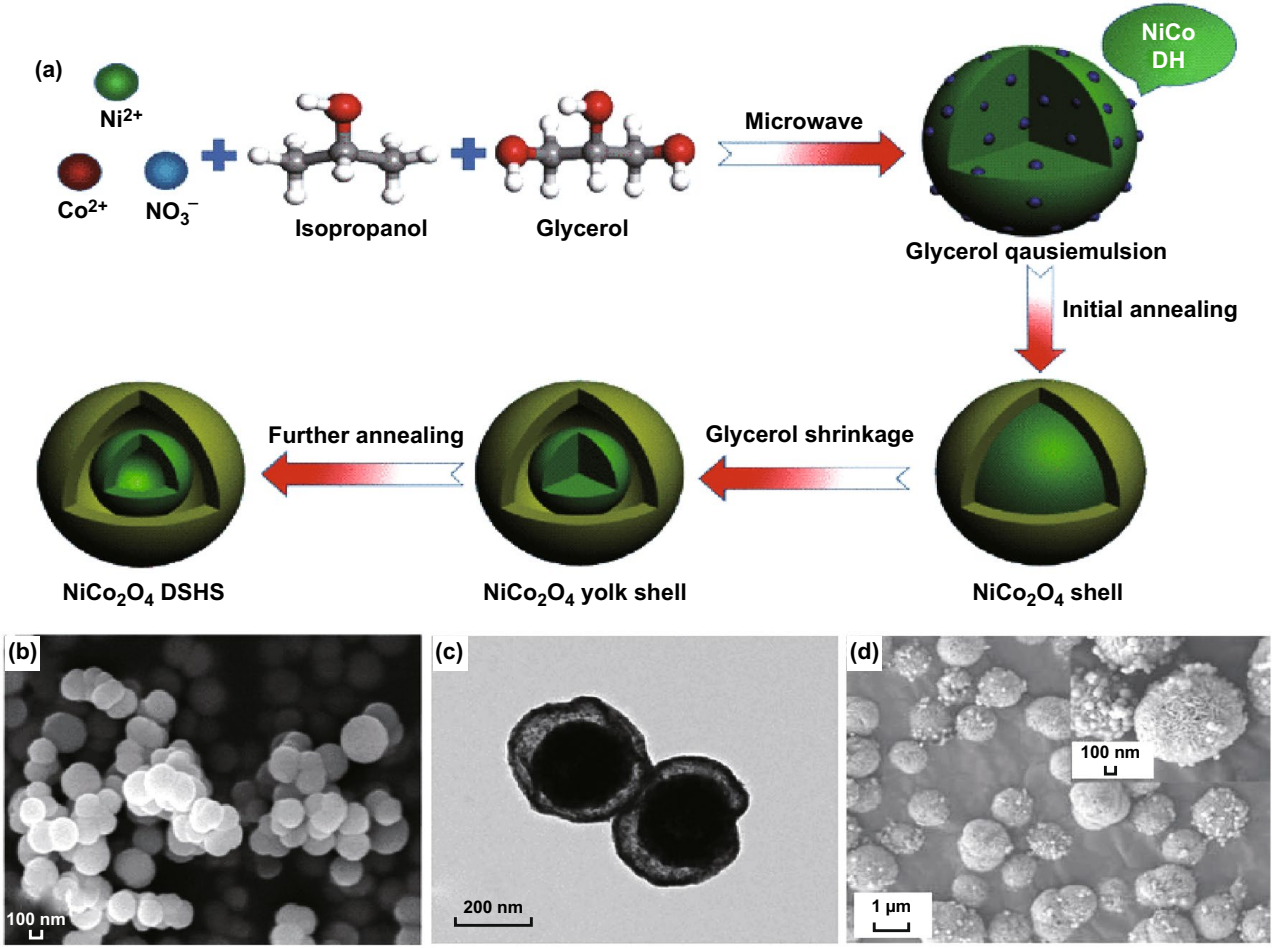
**a** Pictorial representation of the microwave-assisted hydrothermal synthesis of NiCo_2_O_4_ double-shelled hollow spheres. **b** FESEM and **c** TEM images of NiCo_2_O_4_ hollow spheres prepared in the presence of glycerol and d FESEM image of the NiCo_2_O_4_ structures prepared in the absence of glycerol. Reproduced with permission from Ref. [206]. Copyright © 2017 Springer Nature

Microwaves are the electromagnetic radiations having a frequency range between 300 MHz and 300 GHz and a wavelength range of 1 m–1 mm. Microwave-assisted synthesis of nano-/microstructures is superior to the conventional methods described above because it requires a very short reaction duration, is energy efficiency, cost-effectiveness, and gives an excellent yield of highly porous materials. Microwaves result in volumetric heating as they can penetrate throughout the volume of reactants [204]. This volumetric heating is caused by various types of polarization in the medium, including electron polarization, atomic polarization, directional polarization, and space charge polarization [205]. To obtain better morphological results, microwave-assisted synthesis of nanomaterials is usually combined with other synthetic methods such as sol–gel, co-precipitation, and hydro/solvothermal, etc. Recently, the improvement in the hydrothermal method in harmony with microwave assistance has been studied to synthesize NiCo_2_O_4_ nano-/microstructures. Other ways of engineering the structural aspects of the NiCo_2_O_4_ are the use of a template, capping agents, organic solvents, ionic solvents, and addition of other growth additives. The microwave-assisted hydrothermal method was applied by Zhang et al. [206] to prepare NiCo_2_O_4_ double-shelled hollow spheres with an outer and inner shell thickness of ~ 20 and ~ 70 nm, respectively. A mixture of isopropanol and glycerol was used to prepare a reaction solution (Fig. 19a). Glycerol molecules were supposed to form a self-assembled quasi-emulsions in isopropanol that serve as a soft template for the growth of Ni–Co double hydroxides. In the absence of glycerol, solid microspheres with diameters of ~ 1 µm were formed, demonstrating the templated role of glycerol in the synthesis of a double-shelled hollow nanostructure (Fig. 19b–d). In the presence of microwaves, the reaction mixture is heated due to dielectric loss, which significantly accelerates the reaction kinetics. Additionally, the presence of microwaves improves uniformity in terms of dispersion and size distributions.

**Fig. 20:**
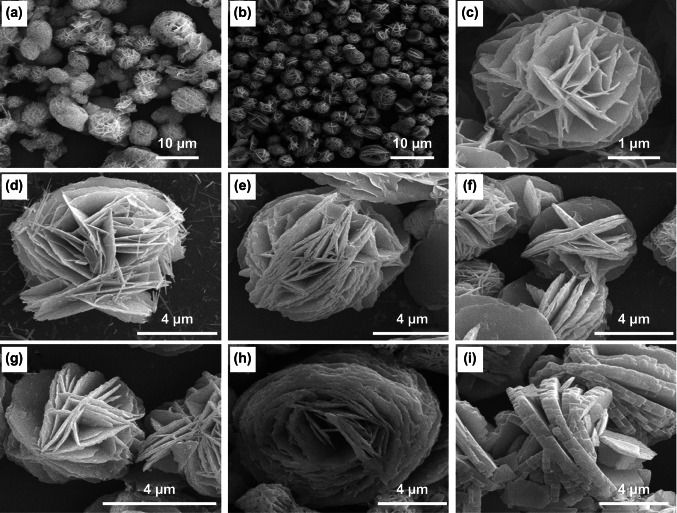
FESEM images of bimetal Ni-Co-precursors obtained under various microwave-assisted hydrothermal reaction times. **a** 5 min, **b, c** 10 min, **d** 15 min, **e** 20 min, **f** 25 min, **g** 30 min, **h** 35 min, and **i** 40 min. Reproduced with permission from Ref. [103]. Copyright © 2019 Elsevier Inc.

Shanmugavani et al. [207] analyzed the effect of reaction times on the morphology of the NiCo_2_O_4_/NiO nanocomposites. The reaction was carried out in the presence of oxalic acid at an operating frequency of 2.45 GHz and 800 W output power. It was proposed that the initially formed nanoparticles are converted into bundled-like structures as the reaction time was increased. Recently, Sun et al. [103] reported novel porous nanoscale NiO/NiCo_2_O_4_ heterostructure through two-stage calcination of nickel–cobalt bimetallic hydroxide precursors (NiCo precursors) which were initially synthesized using a microwave-assisted hydrothermal method in the presence of HMTA and NH_4_F. Notably, F^−^ ions were supposed to act as functional template agents. Prolonged irradiation significantly affects the morphology of NiCo_2_O_4_ materials. When the irradiation time was increased from 5 to 40 min, the incompletely self-assembled and non-uniform 2D nanosheets are converted into more optimized and thickened 3D frameworks with large open spaces (Fig. 20a–i).

Nakate et al. [208] prepared nanocrystalline NiCo_2_O_4_ nanoplates in the surfactant-free environment using metal chloride salts precursors through microwave irradiation. Gu et al. [209] reported 3D nanosphere-like NiCo_2_O_4_ nanostructure composed of intertwined 2D ultrathin mesoporous nanosheets having large specific surface area 146.5 m^2^ g^−1^. The reaction solution was exposed to microwaves (power 560 W) for 6 min. Su et al. [210] reported highly crystalline NiCo_2_O_4_ supported on carbon black via a simple, one step intermittent microwave heating method avoiding the calcination process. However, in a contrary study, Tao et al. [211] analyzed the effect of post-annealing temperature on the morphologies of the NiCo_2_O_4_. Ni–Co double hydroxide was initially prepared through a microwave-assisted method using a tertbutanol solution (98%). Flower-shaped morphology of the Ni–Co double hydroxide was completely converted into unique coral-like morphology on calcination. As the post-annealing temperature was increased from 400 to 700 °C, individual ultrathin nanosheets shrink to smaller nano-sized crystal grains which finally self-assembled to form coral-like NiCo_2_O_4_ architectures.

For greener perspectives, ionic solvents like [1-butyl-3-methylimidazolium][BF_4_] {[Bmim][BF_4_]}, [Bmim]FeCl_4_, [Bmim]Cl [212], and non-ionic glucose-based polymeric surfactant, β-C_10_Alkyl Poly Glucoside [213] are also reported in the literature for the synthesis of NiCo_2_O_4_ architectures with versatile morphologies.

### Spray Pyrolysis Method

**Fig. 21:**
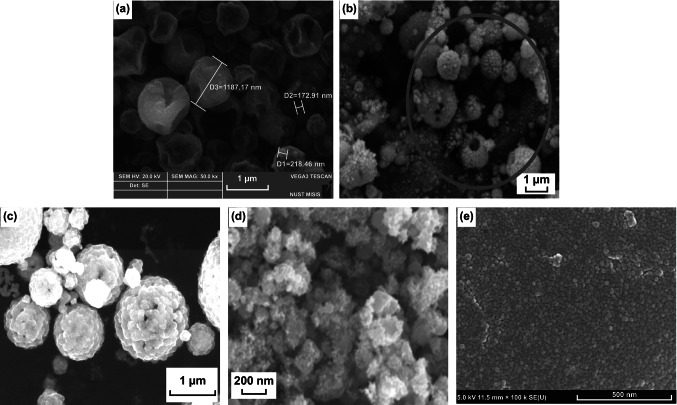
Morphologies of various NiCo_2_O_4_ nanostructures **a** hollow nanosphere. Reproduced with permission from Ref. [215]. Copyright © 2017 The Korean Society of Industrial and Engineering Chemistry, Published by Elsevier B.V. **b** hollow microspheres. Reproduced with permission from Ref. [216]. Copyright © 2019 Elsevier Ltd and Techna Group S.r.l. **c** yolk–shell microspheres. Reproduced with permission from Ref. [218]. Copyright © 2017 Elsevier Ltd. **d** nanoaggregates. Reproduced with permission from Ref. [219]. Copyright © 2015 Elsevier Inc. and **e** thin films with uniform particle distribution size 20–30 nm. Reproduced with permission from Ref. [220]. Copyright © 2016 Elsevier Ltd.

In spray pyrolysis technique, an aerosol of various precursor components is prepared in suitable solvent and is sprayed on the substrate. After that, sequential evaporation of the solvent from the surface of the substrate, heating to precipitate out the solute, high-temperature annealing, formation of microporous particles, and finally, sintering of solid particles is carried out [214]. NiCo_2_O_4_ nanostructures with morphologies hollow nanosphere [215], hollow microspheres [216], dried plum-like particles [217], yolk–shell microspheres [218], nanoaggregates [219], thin films with uniform particle distribution size 20–30 nm [220], etc. are reported (Fig. 21a–e).

**Fig. 22:**
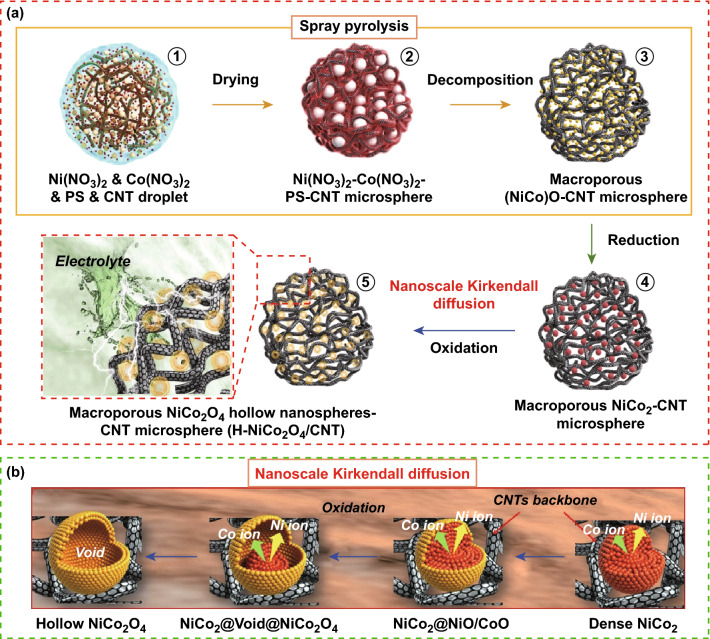
Formation mechanism of 3D macroporous multi-walled carbon nanotubes microspheres densely loaded with NiCo_2_O_4_ hollow nanospheres. Reproduced with permission from Ref. [221]. Copyright © 2017 Elsevier Ltd.

Similar to the electro-spinning method, carbonaceous materials such as reduced graphene oxide, carbon nanotubes, carbon nanofibers are also mixed in the precursor solution to improve the electrochemical properties of NiCo_2_O_4_. Park et al. [221] synthesized three-dimensional macroporous multi-walled carbon nanotubes microspheres densely loaded with NiCo_2_O_4_ hollow nanospheres via spray pyrolysis process. The schematic illustration depicting the formation mechanism is shown in Fig. 22a. The polystyrene nanobeads added in the solution improved the structural uniformity and the dispersion of CNT microspheres. The similarity in the atomic radii of the Ni and Co ions resulted in the Kirkendall diffusion into the outer surface of the where they were oxidized to form NiCo_2_O_4_ (Fig. 22b).

## Biosensor Applications of Nano-/Micro-structured NiCo_2_O_4_

### Glucose Biosensors

Non-enzymatic glucose sensing is considered to be a better, fast, and convenient way as compared to the enzymatic method since the later is a complicated and multi-step process involving immobilization of enzyme bioreceptor such as glucose oxidase, glucose dehydrogenase, and quinoprotein glucose dehydrogenase onto the electrode surface [51, 52]. Furthermore, maintaining the enzyme stability under non-physiological conditions of observations is another major issue related to enzymatic glucose biosensing. Most of the biosensing measurements are based on cyclic voltammetry (CV) and amperometric analysis. Better biosensing behavior and electrochemical activity using NiCo_2_O_4_ nano-/microstructure-modified electrodes are adjudged by broader redox peaks with larger area coverage in the CV curves. Since the spinel NiCo_2_O_4_ comprises binary intrinsic-state redox couples of Ni^3+^/Ni^2+^ (0.58 V/0.49 V) and Co^3+^/Co^2+^ (0.53 V/0.51 V), only a pair of redox peaks in the CV curves is generally observed due to almost similar redox potential values for NiO and Co_3_O_4_ [142, 222, 223]. In alkaline medium, NiCo_2_O_4_ is oxidized to Ni and Co perhydroxides which finally convert glucose into gluconolactone (Eqs. 21–26) [224].

21 $${\text{NiCO}}_{2} {\text{O}}_{4} + {\text{OH}}^{ - } + {\text{H}}_{2} {\text{O}} \leftrightarrow {\text{NiOOH}} + 2{\text{CoOOH}} + {\text{e}}^{ - }$$

22 $${\text{CoOOH}} + {\text{OH}}^{ - } \to {\text{Co}}_{2} {\text{O}}_{3} + {\text{H}}_{2} {\text{O}} + {\text{e}}^{ - }$$

23 $${\text{NiOOH}} + {\text{Glucose}} \to {\text{Ni}}({\text{OH}})_{2} + {\text{Gluconolactone}}$$

24 $${\text{CoOOH}} + {\text{Glucose}} \to {\text{Co}}({\text{OH}})_{2} + {\text{Gluconolactone}}$$

25 $${\text{Ni}}^{2 + } + {\text{Co}}^{2 + } \to {\text{Ni}}^{3 + } + {\text{Co}}^{3 + } + 2{\text{e}}^{ - }$$

26 $${\text{Glucose}} \left( {{\text{C}}_{6} {\text{H}}_{12} {\text{O}}_{6} } \right) \to {\text{Gluconolactone}} \left( {{\text{C}}_{6} {\text{H}}_{10} {\text{O}}_{6} } \right) + 2{\text{H}}^{ + } + 2{\text{e}}^{ - }$$

Since the rates of oxidation of Ni^2+^ and Co^2+^ ions on the electrode surface during anodic scan determine the rate of sensing of glucose, NiCo_2_O_4_ nano-/microstructures with versatile morphologies having large specific surface area, permeability, and most importantly short electron and ion diffusion pathways are synthesized. Ni^3+^ and Co^3+^ ions are reduced back to Ni^2+^ and Co^2+^ ions by the electrons lost by the oxidation of glucose to gluconolactone. According to Hussain et al. [225], H_2_O_2_ is formed as one of the products along with gluconolactone if the electrochemical sensing is performed in the presence of oxygen. Glucose undergoes a spontaneous reaction with water and O_2_ to form gluconolactone which is further oxidized into gluconic acid (Eqs. 27, 28). In a slightly basic medium (pH = 7.4), gluconic acid ionizes to gluconate ions which act as mobile charge carriers on the surface of the NiCo_2_O_4_ nanostructures producing a strong electrical signal (Eq. 29). Elakkiya et al. [226] reported highly porous flower-like NiCo_2_O_4_ nanostructures synthesized via a facile hydrothermal method for excellent electrocatalytic activity in alkaline electrolyte for the oxidation of glucose and lactic acid.272829

**Fig. 23:**
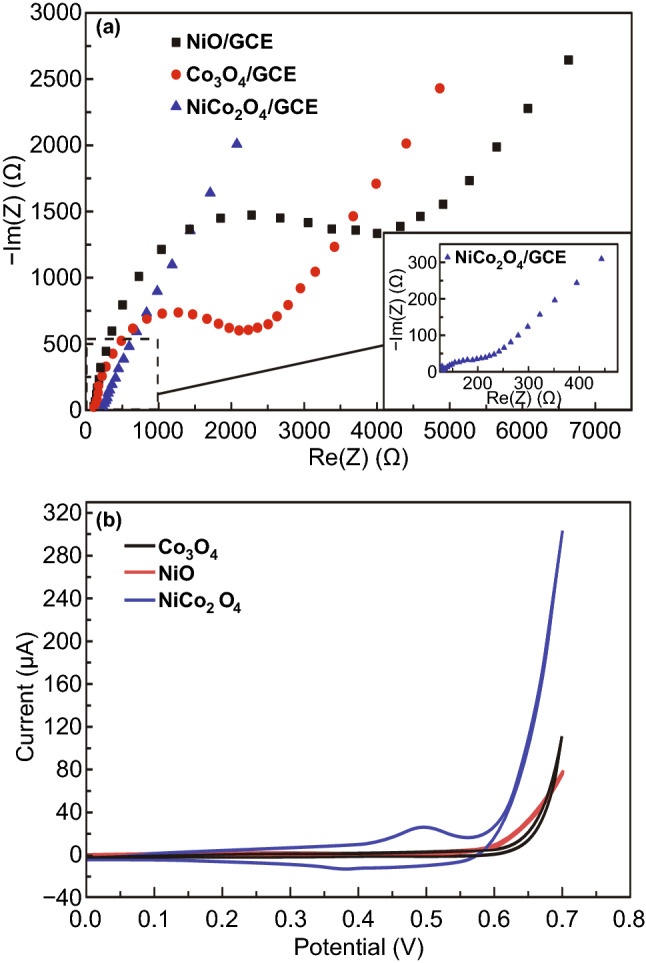
**a** Nyquist plots of NiCo_2_O_4_, Co_3_O_4_, and NiO-modified GCE in 0.1 M NaOH. **b** CV curves for NiCo_2_O_4_, Co_3_O_4_, and NiO-modified GCE in 0.2 M NaOH without glucose. Reproduced with permission from Ref. [140]. Copyright © 2016 Elsevier B.V.

The binary spinel NiCo_2_O_4_ architecture exhibits better intrinsic electronic conductivity as compared to pure NiO and Co_3_O_4_ which is attributed to the doping of Ni^3+^ ions in the octahedral sites of the Co_3_O_4_ crystal lattice which accelerates the electron hopping process [227]. Huang et al. [140] compared the electron transfer resistance (*R*_et_) through electrochemical impedance spectroscopy for GCE modified with NiCo_2_O_4_, NiO, and Co_3_O_4_. Nyquist plots for all the modified GCE consisted of two portions; an inclined line at low frequencies and a semicircular portion at high frequencies. However, the lowest *R*_et_ of NiCo_2_O_4_/GCE was an indication of the enhanced conductivity for NiCo_2_O_4_ (Fig. 23a). Broader redox peaks NiCo_2_O_4_/GCE as compared to NiO/GCE and Co_3_O_4_/GCE confirmed the better biosensing behavior of the NiCo_2_O_4_ as compared to Co_3_O_4_ and NiO (Fig. 23b).

Spinel NiCo_2_O_4_ hollow nanocages were prepared by using Co-based zeolite imidazole frameworks (ZIF-67) as a template and precursor by Feng et al. [228]. Morphological characterization revealed that the thickness of the cage shell was about 30 nm. The outer surface of the nanocages was covered with small nanosheets. A wide linear dynamic range 0.18 μΜ–5.1 mM, high sensitivity 1306 μA mM^−1^ cm^−2^, a fast response time of 1 s, and limit of detection 27 nM were observed for NiCo_2_O_4_ hollow nanocage-based modified GCE.

**Fig. 24:**
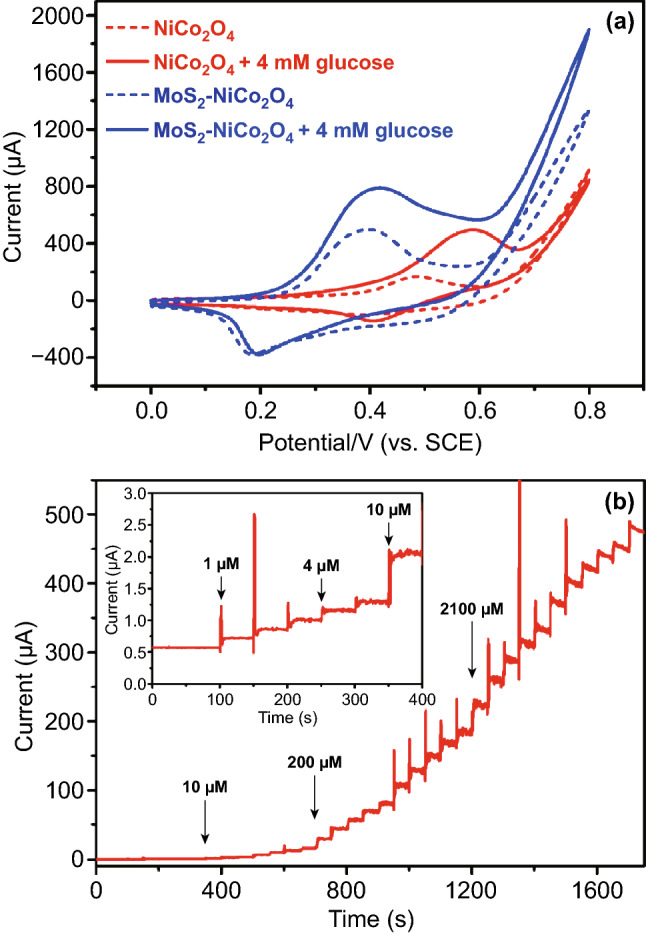
**a** Electrocatalytic activities of MoS_2_-NiCo_2_O_4_/GCE in 0.1 M NaOH at a scan rate of 50 mV s^−1^. **b** Amperometric response curves for MoS_2_-NiCo_2_O_4_/GCE. Reproduced with permission from Ref. [229]. Copyright © 2017 Elsevier B.V.

NiCo_2_O_4_ nanoplates interconnected through MoS_2_ nanosheets performed excellent electrocatalytic behavior toward glucose. NiCo_2_O_4_ nanoplates and MoS_2_ nanosheets illustrated a significance synergic effect. Though not an active catalyst for the oxidation of glucose, the highly active edge of vein-like MoS_2_ nanosheets inhibited the agglomeration of NiCo_2_O_4_ nanoplates and formed long conducting chains which provide an alternative pathway with lower electrical resistance [229] (Fig. 24a, b). The fabricated glucose biosensor exhibited a high sensitivity of 1748.58 μA mM^−1^ cm^−2^ and a very low detection limit of 0.152 μM. MoS_2_ nanosheets have also been reported as support material for the fabrication of NiCo_2_O_4_/MoS_2_ nanocomposites through a simple ionothermal method in deep eutectic solvent (choline chloride (ChCl)-urea mixture) [230]. Deep eutectic solvents consist of simple eutectic-based ionic liquids prepared by eutectic mixing of ChCl and some hydrogen bond donors like acids, amides, alcohols, etc. [231]. These solvents have excellent thermal stability, high surface tensions, negligible vapor pressure, and most importantly biodegradability [232–236]. The NiCo_2_O_4_-MoS_2_/chitosan/GCE-modified electrode was used as an electrochemical sensor for glucose in red wine and honey [230].

**Fig. 25:**
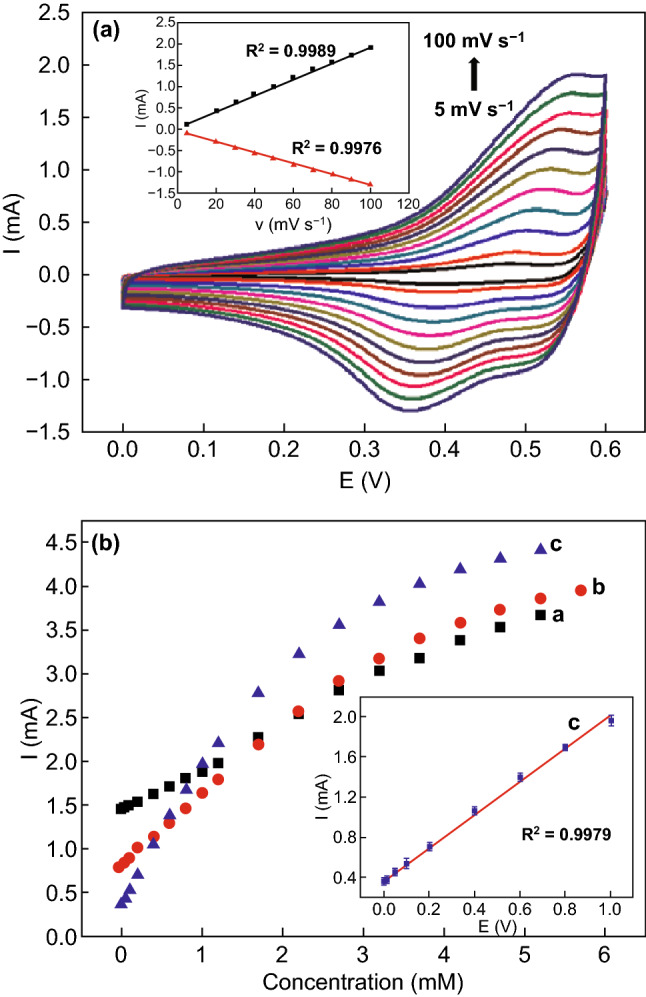
**a** CV curves of NiCo_2_O_4_ hollow nanorods with various scan rates. Inset: redox peak currents as a function of the scan rates, **b** variation in response current for NiO (curve a), Co_3_O_4_ (curve b), and NiCo_2_O_4_ hollow nanorods (curve c) as a function of glucose concentrations. Inset: calibration curve of NiCo_2_O_4_ hollow nanorods. Reproduced with permission from Ref. [142]. Copyright © 2015 Elsevier B.V.

Analysis of non-enzymatic glucose sensing properties of NiCo_2_O_4_ nanosheets showed linear response with respect to the change in glucose concentration varying from 5 to 65 μM. The high sensitivity of 6.69 μA μM^−1^ cm^−2^ with a LOD value of 0.38 μM and liquid of quantification of 1.27 μM was observed. During CV measurements, scan rates increased the oxidation and reduction peak currents as well as peak-to-peak separations [224]. The electrochemical kinetics of the NiCo_2_O_4_ hollow nanorods grown on stainless steel via a sacrificial template showed similar trends during glucose sensing in 0.1 M NaOH solution with scan rates ranging from 5 to 100 mV s^−1^ (Fig. 25a). Amperometric studies revealed a steady-state current optimization within 2 s of glucose addition. Calculated sensitivity, linear detection range, and detection limit were 1685.1 µA mM^−1^ cm^−2^, 0.0003–1.0 mM, and 0.16 µM (S/N = 3), respectively (Fig. 25b) [142]. Cui et al. [237] prepared rectangular flake-like mesoporous NiCo_2_O_4_ via a facile hydrothermal method and observed glucose biosensing sensitivity of 662.31 µA mM^−1^ cm^−2^ and very low detection limit of 0.3 nM at S/N = 3. The other optimized operational parameters were: 0.2 M KOH, + 0.5 V applied potential and 1.0 mg mL^−1^ loading of meso-NiCo_2_O_4_ in the suspensions. Dry rod-like NiCo_2_O_4_ synthesized through a facile hydrothermal reaction followed by subsequently microwave treatment. The non-enzymatic glucose sensor fabricated using these rod-like features showed a high sensitivity of 431.29 µA mM^−1^ cm^−2^ [238]. The microwave treatment completely removed the water and made the material highly porous for exhibiting excellent biosensing applications. One-dimensional porous NiCo_2_O_4_ nanowires array grown on nickel foam (NiCo_2_O_4_ NWs/NF) via a facile hydrothermal method exhibit highly efficient glucose sensitivity of 5916 μA mM^−1^ cm^−2^, a detection limit of 1 μM–3.987 mM and LOD of 0.94 μM (S/N  =  3) [239]. As conducting substrate, nickel foam not only provides the large electrochemically active surface area due to three-dimensional interconnected features, but also directs the growth of one-dimensional NiCo_2_O_4_ porous nanowires [240]. Besides, the one-dimensional porous NiCo_2_O_4_ nanowires array provided sufficient transport channels for ions and abundant active sites for redox reactions. Carbon cloth has also been used as a potential conducting surface for the growth of porous NiCo_2_O_4_ nanowires. As fabricated enzyme-free NiCo_2_O_4_ porous nanowire arrays supported on carbon cloth-based electrode for glucose sensing exhibited a linear dynamic range of 1 μM–0.63 mM, the sensitivity of 4.12 mA mM^−1^ cm^−2^, and low detection limit of 0.5 μM [241].

One of the main disadvantages of using bare NiCo_2_O_4_ is its poor electrical conductivity. However, this limitation can be overcome by forming its composite/hybrid materials. It has been reported that the electrical conductivity and hence the electrochemical biosensing performance of NiCo_2_O_4_ can be improved by making its composites with conducting carbonaceous materials like graphene, reduced graphene oxide, carbon nanotubes (single and multi-walled), carbon nanofibers; conducting polymers like polypyrrole (PPy), polyaniline (PANI); metal oxides NiO, Co_3_O_4_, SnO_2_, MnO_2_; and metals like Au, Pd, etc. Among these, the carbonaceous materials are considered to be potential candidates as compared to others due to their excellent electrical conductivities, good mechanical strength, thermal and chemical stabilities, and resistance to oxidation–reduction reactions. Besides, these carbonaceous materials provide a large specific surface area for better adsorption of analytes, which ultimately results in very high sensitivity and very low detection limits.

**Fig. 26:**
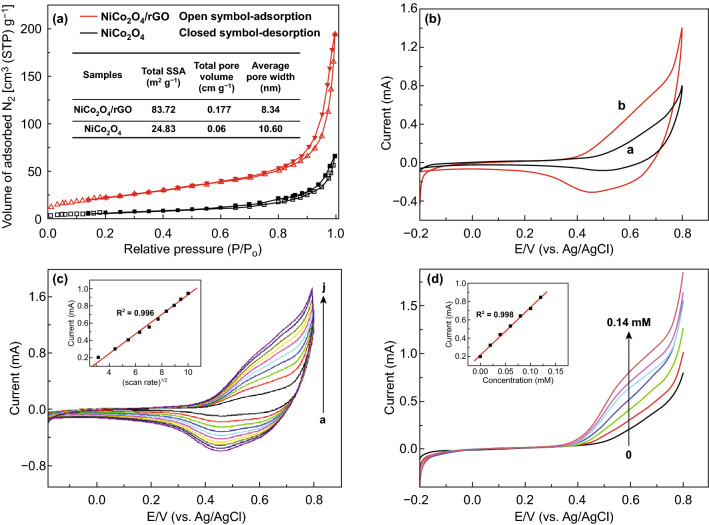
**a** Adsorption–desorption hysteresis loop, specific surface area (SSA), average pore width, and total pore volume of the synthesized pure NiCo_2_O_4_ and NiCo_2_O_4_/graphene nanohybrids. **b** CV for NiCo_2_O_4_/graphene hybrid-modified electrode. **c** Effect of scan rates scan rate for the solution with 0.1 mM glucose in 0.1 M NaOH. **d** Linear sweep voltammetric curves for glucose in the concentration of 0–0.14 mM and calibration plot (Inset). Reproduced with permission from Ref. [244]. Copyright © 2016 Elsevier B.V.

The two-dimensional one-atom-thick layered structure of graphene has been extensively used for making composites with NiCo_2_O_4_ due to its high specific surface area of 2670 m^2^ g^−1^ and excellent conductivity [242, 243]. Studies have revealed a higher specific surface area for the NiCo_2_O_4_/reduced graphene oxide composites as compared to bare NiCo_2_O_4_ nanoparticles (Fig. 26a) [244]. Even the pore width was less in the case of NiCo_2_O_4_/reduced graphene oxide composites. Various glucose-sensing scans are given in Fig. 26b–d. The enhanced redox peak current density for NiCo_2_O_4_/reduced graphene oxide composites as compared to pure NiCo_2_O_4_ was attributed to the lesser extent of aggregation of graphene sheets due to the interception of the NiCo_2_O_4_ nanoparticles on graphene surface causing weakening of *π*–*π* interaction between individual graphene sheets, faster diffusion rates and electron transfer between the glucose molecules and the electrode surface [245].

**Fig. 27:**
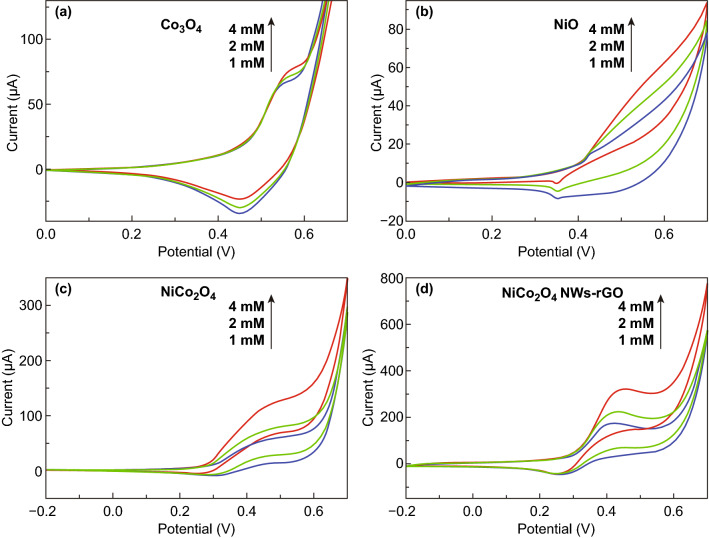
CV plots for different glucose concentrations (1–4 mM) in 0.1 M NaOH **a** Co_3_O_4_, **b** NiO, **c** NiCo_2_O_4_, and **d** NiCo_2_O_4_ nanowrinkles/reduced graphene oxide-modified GCE. Reproduced with permission from Ref. [246]. Copyright © 2016 Elsevier Ltd.

Ma et al. [246] developed NiCo_2_O_4_ nanowrinkles/reduced graphene oxide hybrid-based modified GCE for non-enzymatic glucose detection at the physiological level. As far as the concentration of the glucose is concerned, the oxidation potential of glucose decreased while oxidation peak current increased proportionally to a greater extent for NiCo_2_O_4_ nanowrinkles/reduced graphene oxide hybrid-based modified GCE as compared to single component Co_3_O_4_, NiO and bare NiCo_2_O_4_ at a scan rate of 100 mV s^−1^ in 0.1 M NaOH (Fig. 27a–d). The results confirmed the crucial role of reduced graphene oxide in improving the electrocatalytic biosensing performance of the NiCo_2_O_4_ spinel for different concentrations of glucose.

**Fig. 28:**
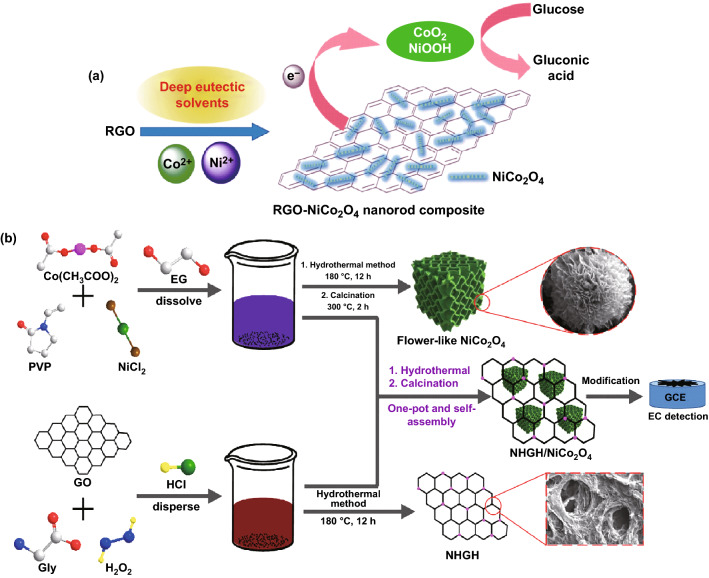
**a** Proposed mechanism of glucose sensing using NiCo_2_O_4_ nanorods/rGO/Nafion composite-modified GCE. Reproduced with permission from Ref. [247]. Copyright © 2018 Elsevier Ltd. **b** Schematic presentation of the synthesis of the NHGH/NiCo_2_O_4_ electro-catalyst for non-enzymatic glucose sensing, Reproduced with permission from Ref. [38]. Copyright © 2019 Elsevier B.V.

In addition to two-dimensional graphene, Wu et al. [245] reported the synthesis of three-dimensional graphene foam (3DGF) through a chemical vapor deposition technique. The 3DGF provides additional stability and large porous surface as well as high conductivity to the hierarchical NiCo_2_O_4_ composites. NiCo_2_O_4_ hierarchical nanoneedles were deposited onto the surface of 3DGF via a hydrothermal method. The synergism between hierarchical NiCo_2_O_4_ nanoneedles and 3DGF exhibited a high sensitivity of 2524 μA mM^−1^ cm^−2^ and a limit of detection 0.38 μM (S/N = 3). Further, as fabricated electrode showed excellent selectivity for glucose even in the presence of interfering compounds like dopamine, ascorbic acid, lactose, d-Fructose, and urea as negligible current responses were observed on their additions as compared to glucose. NiCo_2_O_4_ nanospheres/reduced graphene oxide composite prepared by a template-based method using the Cu_2_O/GO template achieved a high sensitivity of 2082.57 μA mM^−1^ cm^−2^, the detection range of 0.04–1.28 mM, and low detection limit of 0.7 μM [137]. Ni et al. [247] reported a reduced graphene oxide supported NiCo_2_O_4_ nanorods composite prepared via an ionothermal method using deep eutectic solvents. The modified GCE exhibited superior electrocatalytic biosensing of glucose with a wide double-linear range from 1 μM to 25 mM and a very low detection limit of 0.35 μM (S/N  =  3). The presence of a large number of small interconnected nanoparticles on the surface of the NiCo_2_O_4_ nanorods provided the dense electrocatalytic active site in coordination with reduced graphene oxide which provided large surface area and excellent electrical conductivity (Fig. 28a).

Another way of preventing the aggregation of graphene sheets, which reduces the specific surface area and inhibits the fast mass transfer, is the nitrogen doping. This nitrogen doping is not only supposed to facilitates the charge transfers between adjacent carbon atoms but also suppresses the electrons and holes recombination necessary for better electrical conductivity and electrocatalytic oxidation of glucose [248, 249]. Detailed characterization revealed that in the course of hydrothermal reactions, the graphene was reduced to nitrogen-doped reduced graphene oxide when glycine acted as a source of nitrogen. Further, the nitrogen-doped reduced graphene was self-assembled into hydrogels with interconnected 3D porous network structure resulted from an increased extent of *π*–*π* stacking interactions. This 3D form provides a sufficiently large surface area and active sites for the better adsorption of the analyte species. To ascertain this, Lu et al. [38] explored the interactions of flower-like NiCo_2_O_4_ and 3D nitrogen-doped holey graphene hydrogel (NHGH)-modified GCE for electrochemical biosensing of glucose (Fig. 28b).

Similar to graphene, carbon nanofibers also possess excellent dimensional, thermal and chemical stability as well as good electrical conductivity. Recently, these fibers have attracted wide attention and have been widely explored in fields such as electrochemical cells, catalysis, adsorption, structure enhancement, biosensors, gas sensors, and nanodevices [250, 251]. Among various synthetic methods, electro-spinning is considered to be the most suitable low-cost and simple method for synthesizing carbon nanofibers [252, 253]. Liu et al. [39] explored the glucose-sensing behavior of NiCo_2_O_4_ nanoneedle-decorated electrospun carbon nanofiber nanohybrids. Faster electrocatalytic oxidation of glucose was reported for nanohybrids as compared to bare NiCo_2_O_4_ nanoneedle and electrospun carbon nanofiber-modified GCEs. The fact was supported by a large increase in the anode peak current and a positive shift in the anode peak potential.

Novel metals such as Au, Ag, and Pd, have also been used to prepare NiCo_2_O_4_ composites to improve the biosensing capabilities. Recently, dealloying has been used as a convenient method for preparing nanoporous metals with a 3D bicontinuous structure, which is characterized by open nanopores with adjustable sizes [254–256]. These 3D nanoporous metals act as conductive surfaces for the deposition of biosensors electrocatalytic materials such as NiCo_2_O_4_ since they provide high conductivity and large surface area. Disposable needle-type hybrid electrode comprising a stainless steel core modified with a 3D nanoporous Au/NiCo_2_O_4_ nanowall hybrid structure-modified electrochemical non-enzymatic glucose sensor showed a linear response of 0.01–21 mM glucose, high sensitivity of 0.3871 μA μM^−1^ cm^−2^, detection limit of 1 μM within a response time of < 1 s [257]. Naik et al. [258] compared the bare NiCo_2_O_4_/Ni foam, NiCo_2_O_4_–Ag/Ni foam and NiCo_2_O_4_–Au/Ni foam nanosheets electrodes. The calculated sensitivity for pure NiCo_2_O_4_, NiCo_2_O_4_–Ag, and NiCo_2_O_4_–Au nanosheets electrodes in the linear range 5–45 μM and 45–465 μM were 20.8, 29.86, and 44.86 μA μM^−1^ cm^−2^ and 6.2, 11.5, and 13.96 μA μM^−1^ cm^−2^, respectively. The respective limits of detection were 9.33, 5.82, and 2.64 μM. DFT studies confirmed strong binding between Au and NiCo_2_O_4_ as compared to Ag. Further, the binding energy of glucose was more for the NiCo_2_O_4_–Au surface compared to the NiCo_2_O_4_–Ag surface. The enhanced density of states near the Fermi level improved the conductivity of the NiCo_2_O_4_–Au nanosheet than NiCo_2_O_4_–Ag that caused superior glucose sensing performance. In a similar type of report, the sensitivities for pure NiCo_2_O_4_ and NiCo_2_O_4_–Pd nanosheets electrodes in the linear range 5–90 μM and 70–450 μM were 27.5 and 40.03 μA μM^−1^ cm^−2^ and 8.53 and 8.23 μA μM^−1^ cm^−2^, respectively [259].

**Fig. 29:**
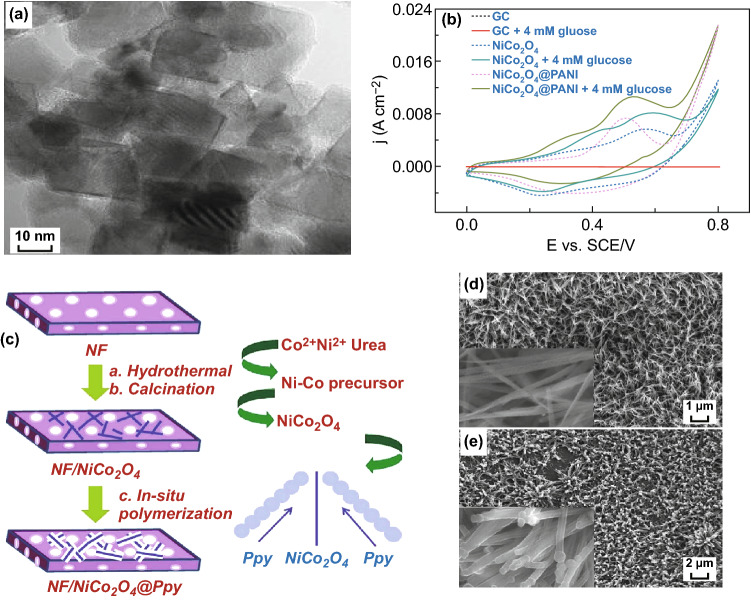
**a** HRTEM image of NiCo_2_O_4_@PANI nanocomposite and **b** CV plots for different GCE electrodes in the absence and presence of 4 mM glucose in 0.1 M NaOH solution at scan rate = 50 mV s^−1^. Reproduced with permission from Ref. [263]. Copyright © 2015 Elsevier B.V. **c** Schematic illustration of the preparation of core–shell NiCo_2_O_4_@Ppy on Ni foam substrate. **d** FESEM image of bare NiCo_2_O_4_. **e** FESEM image of NiCo_2_O_4_@Ppy composite, Reproduced with permission from Ref. [264]. Copyright © 2019 Elsevier B.V.

Similar to metals, conducting polymers also possess the electronic, electrical, and optical properties, easy synthesis, excellent mechanical stabilities and most importantly the low toxicity and biodegradability, the issues which are generally associated with metals. Moreover, the noble metals are easily poisoned by some intermediates produced during the oxidation of glucose. Among various conducting polymers, polyaniline and polypyrrole have gained much attention due to their superior thermal and oxidative stabilities [260, 261]. Constructing a core–shell nanostructure comprising conductive polymer coating as the outer walls of metal oxides is the most important strategy for enhancing the conductivities [262]. NiCo_2_O_4_@PANI nanoparticles with an average particle size 25 nm shortened the ion transport pathway and the modified GCE exhibited a sensitivity of 4.55 mA mM^−1^ cm^−2^, a detection limit of 0.3833 μM and linear dynamic range of 0.0150–4.7350 mM (Fig. 29a, b) [263]. The PANI core–shell provided more effective electrical contact between redox-active centers and the electrolyte resulting in good contact and small diffusion distances for electron transports which subsequently improved the sensor activity. NiCo_2_O_4_@Ppy nanowires grown on Ni foam were synthesized via hydrothermal growth and oxidant-induced polymerization process (Fig. 29c–e). The fabricated glucose sensor showed high sensitivity 3059 μA mM^−1^ cm^−2^, low detection limit 0.22 μM, and wide linear dynamic range 0.001–20 mM. The excellent electrocatalytic behavior was attributed to the synergism due to bimetallic oxide, the significant role of Ppy in transmitting charges among electrode material due to its excellent conductivity, non-collapsing and non-agglomeration of the NiCo_2_O_4_ due to Ppy coating, and absence of any adhesive or conductive agent during electrode fabrication [264].

NiCo_2_O_4_ nano-/microstructures combined with smarter nano-architectured metal oxides (Co_3_O_4_, SnO_2_, NiO, and MnO_2_, etc.) have many synergistic multifunctional properties of nanostructured components including dense and easily accessible electroactive sites, altered bandgap energies, faster charge transfer processes, and reduced internal resistance. The p-type semiconductor nanostructured NiCo_2_O_4_ [32], Co_3_O_4_ [265], and n-type materials SnO_2_ [266] and MnO_2_ [267] have the bandgap energies of 2.1, 2.2, 3.6, and 1.3 eV, respectively. Due to slightly different bandgap energies, the semiconductor metal oxides introduce in situ impurity bands in NiCo_2_O_4_ which increase the electron conductivity to extract excellent electrocatalytic efficiencies [268]. Chen et al. [269] reported porous Co_3_O_4_ nanosheets synthesized via a simple hydrothermal method. The Co_3_O_4_ nanosheets provided the growth sites for the hydrothermal synthesis of NiCo_2_O_4_ nanorods. At the optimized conditions, porous Co_3_O_4_–NiCo_2_O_4_ nanosheet-modified GCE exhibited a preeminent sensitivity of 1463.13 μA mM^−1^ cm^−2^, a low detection limit of 0.112 μM and linear dynamic range 0.001–2.1 mM with excellent selectivity and reproducibility. The amperometric current–time plot showed a successive increase in current with the concentration of glucose (1 μM–6.1 mM) at an applied voltage of + 0.55 V using porous Co_3_O_4_–NiCo_2_O_4_ nanosheet-modified GCE. The current–concentration calibration plot displayed two linear portions with concentration ranges 1 μM–2.1 mM and 2.1–6.1 mM. Further, the incorporation of graphene into Co_3_O_4_/NiCo_2_O_4_ double-shelled nanocages was explored by Xue et al. [270]. The Co_3_O_4_/NiCo_2_O_4_ double-shelled nanocages were prepared by using zeolite imidazole frameworks-67 as a template. The amperometric studies revealed a sensitivity of 0.196 mA mM^−1^ cm^−2^ with detection limit 0.744 μM in linearized concentration range 0.01–3.52 mM, whereas, in linearized concentrations range of 0.01–3.52 mM, the sensitivity was 0.304 mA mM^−1^ cm^−2^ with detection limit 0.384 μM.

The introduction of n-type semiconductors stuffs like SnO_2_ in p-type NiCo_2_O_4_ semiconductors results in the formations of n–p junctions that facilitate the photo-induced electrochemical changes by altering the bandgap energies. Cai et al. [118] observed a prompt photocurrent reduction with the addition of the 100 µL–20 mM glucose solutions into the electrolyte solution. It was proposed that under sunlight stimulation, electron–hole (e^−^–h^+^) pairs are generated by the excitation of the electrons from the valence band of the n-type SnO_2_ semiconductor after the absorption of light of suitable energy (more than bandgap energy). The OH^−^ of the solid electrolyte trap these h^+^ holes and form ${\text{OH}}^{ \cdot }$ radicals (Eq. 30). The ${\text{OH}}^{ \cdot }$ radicals are then transferred to the counter electrode to oxidize NiCo_2_O_4_ to NiOOH and CoOOH (Eq. 31). However, in the presence of glucose, positively charged h^+^ causes oxidation of glucose to gluconolactone. The electrons released during the oxidation process are transferred back to the valence band, so the photocurrent is decreased.

30 $${\text{OH}}^{ - } + {\text{h}}^{ + } \to {\text{OH}}^{ \cdot }$$

31 $${\text{NiCo}}_{2} {\text{O}}_{4} + {\text{OH}}^{ \cdot } + {\text{H}}_{2} {\text{O}} \to {\text{NiOOH}} + {\text{CoOOH}}$$

Chen et al. [271] synthesized bionics-inspired streptococcus-like mixed oxide NiCo_2_O_4_ coated on needle-like MnO_2_ architectures. Initially, MnO_2_ nanowires were synthesized via a quick precipitation method, while NiCo_2_O_4_ were grown on pre-synthesized MnO_2_ nanowires via a facile hydrothermal method. MnO_2_ nanowires prevented the agglomeration of NiCo_2_O_4_ by acting as nucleation sites and electrocatalytic centers for the uniform growth of NiCo_2_O_4_. The synergism between NiCo_2_O_4_ and MnO_2_ was explored for the non-enzymatic electrochemical sensing of glucose. NiCo_2_O_4_–MnO_2_/GCE exhibited high sensitivity, wide concentration ranges, very low detection limit, and long-term stability as compared to NiCo_2_O_4_/GCE and MnO_2_/GCE.

Numerous studies have been conducted to verify the selectivity of the NiCo_2_O_4_-based modified sensors as ascorbic acid, dopamine, and uric acid coexist along with glucose in human blood serum [272]. Therefore, for practical applications, these components should not affect the amperometric parameters and the positive results have been reported [230, 237, 238, 241, 247, 272, 273]. Additionally, reproducibility and stability of the modified NiCo_2_O_4_-based electrodes have been analyzed. The results have shown acceptable reproducibility with a very low relative standard deviations in many studies [123, 142, 161, 193, 199, 200, 227, 274]. The electrochemical sensing parameters such as sensitivity, linear dynamic range, and detection limits for various NiCo_2_O_4_-based modified electrodes toward glucose are compared in Table 1.

**Table 1:** Electrochemical sensing parameters for various NiCo_2_O_4_-based modified electrodes toward glucose

Sensor material	Sensitivity (μA mM^−1^ cm^−2^)	LDR (mM)	LOD (μM)	Refs.
3D nitrogen-doped holey graphene/NiCo_2_O_4_ nanoflowers	2072.0	0.005–10.95	0.39	[38]
NiCo_2_O_4_/ECF	1947.2	0.005–19.175	1.5	[39]
NiCo_2_O_4_/rGO	2082.6	0.04–1.28	0.7	[137]
Porous NiCo_2_O_4_ hollow nanospheres	1917.0	0.01–2.24	0.6	[140]
Hollow NiCo_2_O_4_ nanorod	1685.0	0.0003–1.0	0.16	[142]
NiCo_2_O_4_ nanosheet	6690.0	0.005–0.065	0.38	[224]
NiCo_2_O_4_ hollow nanocages	1306.0	0.00018–5.1	27^b^	[228]
MoS_2_–NiCo_2_O_4_ architecture	1748.6	1.6–11.1	0.152	[229]
Mesoporous NiCo_2_O_4_	662.3	–	0.3^b^	[237]
Rod-like NiCo_2_O_4_	431.3	–	–	[238]
NiCo_2_O_4_ NWs/NF	5916.0	0.001–3.987	0.94	[239]
NiCo_2_O_4_ NWAs/CC	4.12^a^	0.001–0.63	0.5	[241]
NiCo_2_O_4_ 3DGF	2524.0	0.0005–0.59	0.38	[245]
NiCo_2_O_4_ NWs-rGO	548.9	5–8.6	2.0	[246]
RGO-NiCo_2_O_4_/Nafion/GCE	960.4	0.001–6.3	0.35	[247]
	216.7	6.3–25		
NiCo_2_O_4_ nanowalls/3D nanoporous gold/SS needle	387.1	0.01–21	1.0	[257]
NiCo_2_O_4_@PANI	4550.0	0.015–4.735	0.38	[263]
Co_3_O_4_–NiCo_2_O_4_ nanosheets	1463.13	0.001–2.1	0.112	[269]
Graphene/Co_3_O_4_/NiCo_2_O_4_ DS nanocages	304.0	0.01–3.52	0.384	[270]
NiCo_2_O_4_–MnO_2_ nanosheets	2887.6	0.001–2600	0.036	[271]
Mesoporous NiCo_2_O_4_ nanowires	72.4	0.37–2.0	0.37	[273]
NiCo_2_O_4_ nanorods	4710.0	0.001–0.88	0.063	[276]
Dandelion-like NiCo_2_O_4_ hierarchical microspheres	430.86	10–10480	–	[277]

^a^mA mM^−1^ cm^−2^ units

^b^nM units

**Fig. 30:**
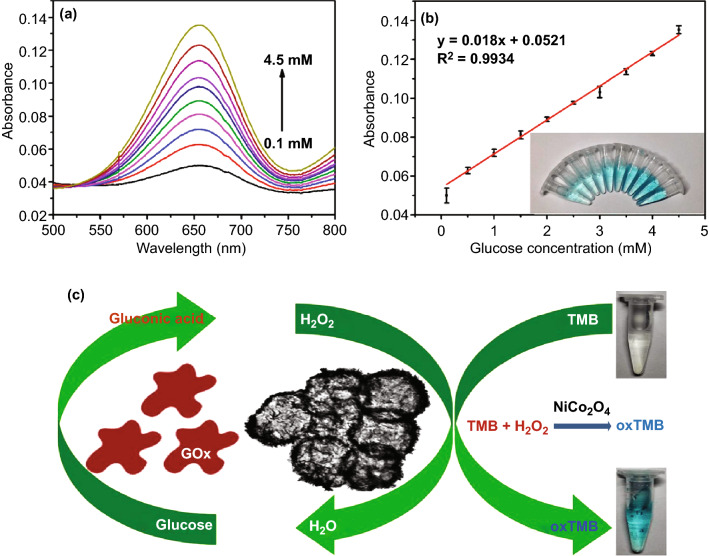
**a** Effect of glucose concentrations on absorption. **b** Linearized calibration curve. **c** Proposed mechanism for colorimetric detection of glucose using the hierarchical NiCo_2_O_4_ hollow sphere. Reproduced with permission from Ref. [274], Copyright © 2017 by the authors; licensee MDPI, Basel, Switzerland

In addition to electrochemical sensing of glucose using NiCo_2_O_4_ nano-/microstructure-modified electrodes, colorimetric sensing has also been reported by Huang et al. [274]. They explored the peroxidase-like activity of the hierarchical NiCo_2_O_4_ hollow sphere which was directly dependent on the concentration of H_2_O_2_ produced by the oxidation of glucose to gluconic acid in the presence of glucose oxidase (Go_x_). Hence, a colorimetric method for the detection of glucose can be designed using NiCo_2_O_4_. The higher the concentration of the glucose, the more was the production of H_2_O_2_ and hence the greater was the oxidation of the 3,3,5,5-tetramethylbenzidine (TMB) to oxidized TMB. Absorbance at *λ*_max_ = 652 nm for oxidized TMB was increased linearly with the concentration of glucose. The linear range was observed between 0.1 and 4.5 mM with a low detection limit of 5.31 μM (Fig. 30a, b). The corresponding reaction mechanism is shown in Fig. 30c. Intrinsic peroxidase and oxidase-like activities of the NiCo_2_O_4_ architectures were also confirmed by Su et al. [275] by analyzing the electron spin resonance spectra for the oxidation of TMB by NiCo_2_O_4_ mesoporous spheres. The oxidation was accompanied without the production of ^1^O_2_ and OH^·^ radicals. Additionally, these peroxidase-like activities were feasible even over a broad temperature range.

### H_2_O_2_ Biosensors

H_2_O_2_ is the most important byproduct produced from some enzyme-catalyzed biochemical reactions. In addition to its importance as a regulator of immune cell activation, vascular remodeling, and stomatal closure during metabolic processes, it also has pharmaceutical, clinical, environmental, textile, and food manufacturing applications [278]. Further, the concentration of H_2_O_2_ in urine is a direct indicator of the whole-body oxidative stress which is the common cause of renal failure, arteriosclerosis, myalgic, encephalomyelitis, Parkinson’s disease, diabetes mellitus, cancer and cardiovascular diseases [279]. Similar to glucose, the literature reports enzymatic as well as non-enzymatic biosensors for the detection of H_2_O_2_. Horseradish peroxidase and heme protein-based enzymatic biosensor are the most researched H_2_O_2_ biosensor due to their high sensitivity, selectivity, and biodegradability. In recent years, non-enzymatic/enzymeless H_2_O_2_ biosensors based on metal oxides have become a new class of biosensors due to fast, low-cost, and easy-to-fabrication processes [280].

In this section of the review, some non-enzymatic H_2_O_2_ biosensors based on NiCo_2_O_4_ spinel nano-/microstructures are discussed. The current–time amperometric H_2_O_2_ biosensing using modified Co_3_O_4_/NiCo_2_O_4_ nanosheets/GCE at an applied potential of − 0.35 V exhibited high sensitivity and low limit of detection of 303.42 μA mM^−1^ cm^−2^ and 0.596 μM, respectively [269]. The Co^3+^ ions of Co_3_O_4_ were supposed to play an important role in the sensing of H_2_O_2_. In alkaline medium, Co^3+^ ions reduce H_2_O_2_ to H_2_O (Eq. 32) [281].

32 $$2{\text{Co}}^{3 + } + {\text{H}}_{2} {\text{O}}_{2} + 2 {\text{OH}}^{ - } \to 2{\text{H}}_{2} {\text{O}} + {\text{O}}_{2} + 2{\text{Co}}^{2 + }$$

The electro-reduction in H_2_O_2_ by NiCo_2_O_4_ spinel-based electrodes occurs according to Eqs. 33–36 [95, 282, 283].333435

36 $$2 {\text{M}}^{{ 3 { + }}} + 2{\text{e}}^{ - } \to 2{\text{M}}^{2 + }$$

Since H_2_O_2_ is reduced to H_2_O (O^2−^ ions) or OH^−^ ions as opposed to oxidation of glucose to gluconolactone, it was found that the current response during electrochemical sensing of H_2_O_2_ was reduced. Similar trends have been reported by other sensor materials such as ZnFe_2_O_4_/reduced graphene oxide [284], nickel phosphide (Ni_2_P) nanosheets array on titanium mesh [285], cobalt nitride (Co_3_N) nanowire array on Ti mesh [286], nanoporous carbon nanofibers/Pt nanoparticles [287], Ag decorated hierarchical Sn_3_O_4_ [288] and many more for the electrochemical sensing of H_2_O_2_ amperometrically.

Xiao et al. [289] reported that NiCo_2_O_4_ mixed oxide-based electrodes in the alkaline medium can cause electro-reduction as well as electro-oxidation toward H_2_O_2_. The extraordinary variety of inter-convertible oxidation states of Co and Ni in spinel NiCo_2_O_4_ is the key factor for its oxidizing and reducing nature. High valence Co^3+^ and Ni^3+^ ions of NiCo_2_O_4_ can be reduced to lower + 2 oxidation states, i.e., into Co^2+^ and Ni^2+^ ions. Similarly, lower + 2 oxidation states can also be oxidized to higher valencies including Co^3+^, Co^4+^, and Ni^3+^ ions. Other factors which decide the electro-reduction and electro-oxidation behavior of the NiCo_2_O_4_ toward H_2_O_2_ are the pH and the concentration of the H_2_O_2_ in the medium. At a scan rate of 10 mV s^−1^, H_2_O_2_ electro-reduction and electro-oxidation were observed for 0.4 M H_2_O_2_ in 3.0 M and 0.75 M H_2_O_2_ in 3.0 M KOH, respectively. Equation 37 represents the overall electro-oxidation of H_2_O_2_ in an alkaline medium.

37 $${\text{H}}_{2} {\text{O}}_{2} + 2{\text{OH}}^{ - } \to {\text{O}}_{2} + 2{\text{H}}_{2} {\text{O}} + 2{\text{e}}^{ - }$$

The electrons lost during the oxidation of H_2_O_2_ reduce the trivalent cations (Co^3+^ and Ni^3+^) ions to their divalent states.

Xue et al. [290] grew ZnO nanowires on Ni foam via a galvanostatic electro-deposition technique. After that, the Ni foam-supported ZnO nanowires and Co_3_O_4_/NiCo_2_O_4_ double-shelled nanocages were prepared by coprecipitation and annealing processes. The ZnO/Co_3_O_4_/NiCo_2_O_4_/Ni foam-based electrochemical H_2_O_2_ sensor exhibited a high sensitivity of 388 μA mM^−1^ cm^−2^, the low detection limit of 0.163 μM, and a dynamic linear range concentration of 0.2 μM–2.4 mM with a fast response time of 5 s. The fast and high response was attributed to the fast electron transport and short electrical pathway provided by ZnO nanowires. Additionally, Co_3_O_4_/NiCo_2_O_4_ double-shelled nanocages provided sufficient mesopores and large specific surface area for improved H_2_O_2_ sensing [290]. Sakthivel et al. [291] compared the electrochemical kinetics of modified NiCo_2_O_4_/GCE, NiCo_2_S_4_/GCE, and NiCoSe_2_/GCE toward H_2_O_2_. The modified NiCoSe_2_/GCE showed better electrochemical sensing behavior for H_2_O_2_ than modified NiCo_2_O_4_/GCE and NiCo_2_S_4_/GCE. The greater electrocatalytic efficiency of modified NiCoSe_2_/GCE was attributed to the large electrochemically active surface area for hydrothermally synthesized NiCoSe_2_.

### Urea Biosensors

Urea (carbamide or carbonyl diamide) is one of the main end products of protein metabolism in humans and animals. Urea is exclusively formed in the liver, and is transported by the bloodstream to the kidneys for excretion in the human body. The normal level of urea in human blood serum is 2.5–7.5 mM [292–295]. Amount of urea above or below the permissible level in the serum results in chronic renal and hepatic failure, gastrointestinal bleeding, and nephritic syndrome [296]. Similar to other metabolically important biomolecules, the literature reports enzymatic as well as non-enzymatic biosensors for the selective and highly sensitive urea sensors. Enzyme-based urea biosensors explore the use of urease enzyme which facilitates the hydrolysis of urea into ammonium (${\text{NH}}_{ 4}^{ + }$) and bicarbonate (${\text{HCO}}_{ 3}^{ - }$) ions (Eq. 38) [297].

38 $${\text{NH}}_{ 2} {\text{CONH}}_{ 2} + 3 {\text{H}}_{ 2} {\text{O}}\mathop{\longrightarrow}\limits^{\text{Urease}} 2 {\text{NH}}_{ 4}^{ + } + {\text{HCO}}_{ 3}^{ - } + {\text{OH}}^{ - }$$

However, in this section, some non-enzymatic-modified urea sensor electrodes based on spinel NiCo_2_O_4_ nano-/microstructures are reviewed. Research has proved that urea can be electrochemically oxidized by NiCo_2_O_4_ nano-/microstructures (Eqs. 39–41).

39 $$6{\text{M}}^{2 + } + 18{\text{OH}}^{ - } \to 6{\text{MOOH}} + 6{\text{H}}_{2} {\text{O}} + 6{\text{e}}^{ - }$$

40 $$6{\text{MOOH}} + {\text{CO}}({\text{NH}}_{2} )_{2} + 2{\text{OH}}^{ - } \to 6{\text{M}}({\text{OH}})_{2} + {\text{CO}}_{3}^{2 - } + \hbox{N}_{2}$$

The overall reaction can be written as:

41 $$6{\text{M}}^{2 + } + {\text{CO}}({\text{NH}}_{2} )_{2} + 20{\text{OH}}^{ - } \to 6{\text{M}}({\text{OH}})_{2} + 6{\text{H}}_{2} {\text{O}} + {\text{CO}}_{3}^{2 - } + {\text{N}}_{2} + 6{\text{e}}^{ - }$$

Recently, Amin et al. [298] explored the urea sensing behavior of NiCo_2_O_4_ nanoneedle-modified GCE which showed high sensitivity with a linear response concentration range of 0.01–5 mM and low detection limit of 1.0 µM. It was proposed that initially Ni^2+^ ions are oxidized to Ni^3+^ ions to form NiOOH in an alkaline medium which is reduced back to give Ni(OH)_2_ at the time of urea electro-oxidation [299]. Mesoporous spinel NiCo_2_O_4_ nanostructures prepared via facile chemical deposition method showed higher catalytic activities, lower overpotential, and more tolerance toward urea electro-oxidation as compared to Co_3_O_4_ [227]. NiCo_2_O_4_/3D graphene/ITO exhibited high sensitivity of 166 μA mM^−1^ cm^−2^, a linear range of 0.06–0.30 mM, and a low detection limit of 5.0 µM with a very small response time of 1 s through non-enzymatic detection method [300]. Further, a higher oxidation peak for NiCo_2_O_4_/3D graphene in the CV as compared to NiCo_2_O_4_/CNT-modified electrode confirmed the superiority of 3D graphene as a matrix material for electrode fabrication. The higher oxidation current potential was attributed to the highly porous nature and excellent conductivity of the interconnected 3D graphene matrix [301]. Since oxidation of urea is in alkaline medium, higher electrocatalytic oxidation of urea is recorded at higher pH conditions. However, beyond an optimum pH the electro-oxidation decreases due to blockage of the active sites by OH^−^ ions [302].

### Electrochemical Determination of Some Other Bioanalytes

Some other bioanalytes such as rutin, trypsin, ascorbic acid, dopamine, uric acid, and tryptophan have also been electrochemically analyzed using nano-/micro-structured hybrid NiCo_2_O_4_-modified electrodes. Rutin, a flavonoid substance, is used as anti-bacterial, anti-viral, antiproliferative, antioxidants, antagonists, and anti-inflammatory. It also controls the blood pressure and vascular fragility including hereditary hemorrhagic telangiectasia, haemangiomas, vitamin C deficiency, etc. [303, 304]. Cui et al. [305] reported the fabrication of GCE modified with mesoporous NiCo_2_O_4_-decorated reduced graphene oxide for the electrochemical sensing of rutin using differential pulse voltammetric (DPV) technique. Flake-like NiCo_2_O_4_ sheets anchored on the wrinkled reduced graphene oxide sheets through electrostatic interaction prevented the self-agglomerations. The wide linear range of 0.1–150 μM and a low detection limit of 0.01 μM were observed along with excellent anti-interference capabilities. The strong synergism between reduced graphene sheets and mesoporous NiCo_2_O_4_ resulted in increased redox peak current and decreased potential difference. During electro-oxidation, rutin is converted into 3′,4′-diquinone with the release of two H^+^ ions and two electrons (Eq. 42) [306, 307].42

Trypsin, a serine protease secreted from the pancreas, has also been widely studied recently as it used for identifying and determining the amino acid sequence in protein and peptide, particularly at the C-terminus and as a specific biomarker for diseases like chronic cystic fibrosis, chronic pancreatitis, cancer, and many pathological changes [308, 309]. Lin et al. [310] reported a large and prompt rise in electrochemical signal in the presence of trypsin by NiCo_2_O_4_- poly(amidoamine)/peptide@g-C_3_N_4_ nanocomposite-modified GCE. 3,4,9,10-perylene tetracarboxylic acid (PTCA) was used to connect the peptides and g-C_3_N_4_. The modified GCE exhibited increased DPVs peak currents when the trypsin concentration was increased from 10^−10^ to 10^−4^ mg mL^−1^. Kaur et al. [311] studied the simultaneous electrochemical sensing of ascorbic acid, dopamine, uric acid, and tryptophan using NiCo_2_O_4_/Nano-ZSM-5 nanocomposite-modified GCE. Wide linear ranges were 1–1200, 0.6–900, 0.9–1000, and 0.9–1000 μM, while the corresponding detection limits were 0.8, 0.5, 0.7, and 0.7 μM for ascorbic acid, dopamine, uric acid, and tryptophan, respectively. Simultaneous detection was possible as the anodic oxidative peak currents were observed at different applied potentials, i.e., 0.158, 0.394, 0.561, and 0.820 V, respectively, for ascorbic acid, dopamine, uric acid, and tryptophan in DVP plots at a scan rate of 20 mV s^−1^.

Detailed comparison from Tables 1 and 2 indicates that the morphology of the NiCo_2_O_4_ nano-/microstructures and the presence of any other component along with NiCo_2_O_4_ significantly affect the biosensing efficiency. Comparative analysis revealed better electrochemical sensing of glucose by one-dimensional nanofibres and nanorods and two-dimensional nanosheets like morphologies of NiCo_2_O_4_ than other morphologies. Doped and composites/hybrid NiCo_2_O_4_ nano-/microstructures exhibit superior sensing parameters that bare NiCo_2_O_4_. In particular, graphenic nanomaterials due to their excellent conductivity and large surface area significantly elevate the *I*–*V* characteristics to many folds. These materials also accelerate the rate of heterogeneous electron transfer, i.e., the transfer of electrons from/to electrode to/from bioanalyte molecules [312].

**Table 2:** Electrochemical sensing parameters for various NiCo_2_O_4_-based modified electrodes toward some bioanalytes

Sensor material	Analyte	Sensitivity (μA mM^−1^ cm^−2^)	LDR (mM)	LOD (μM)	Refs.
3D nitrogen-doped holey graphene/NiCo_2_O_4_ nanoflowers	H_2_O_2_	–	0.001–0.510	0.136	[38]
Co_3_O_4_–NiCo_2_O_4_ nanosheets	H_2_O_2_	303.42	0.02–1.1	0.596	[269]
ZnO/Co_3_O_4_/NiCo_2_O_4_/Ni foam	H_2_O_2_	388.0	0.0002–2.4	0.163	[290]
NiCo_2_O_4_ nanoneedles	Urea		0.01–5	1.0	[298]
Nickel/cobalt oxide-decorated 3D graphene nanocomposite	Urea	166.0	0.06–0.30	5.0	[300]
Mesoporous NiCo_2_O_4_/rGO	Rutin	–	0.1–150	0.01	[305]
NiCo_2_O_4_ nanosheets/g-C_3_N_4_ nanocomposite	Trypsin^a^	–	10^−10^–10^−4^	10^−10 ^	[310]
NiCo_2_O_4_/nano-ZSM-5 nanocomposite	Ascorbic acid	–	1–1200	0.8	[311]
NiCo_2_O_4_/nano-ZSM-5 nanocomposite	Dopamine	–	0.6–900	0.5	[311]
NiCo_2_O_4_/nano-ZSM-5 nanocomposite	Uric acid	–	0.9–1000	0.7	[311]
NiCo_2_O_4_/nano-ZSM-5 nanocomposite	Tryptophan	–	0.9–1000	0.7	[311]

^a^Units in mg mL^−1^

## Conclusion

Herein, various strategies for the synthesis of spinel NiCo_2_O_4_ nano-/microstructures with versatile morphologies and their subsequent use for the development of biosensors for efficient non-enzymatic sensing and detection of biomolecules such as glucose, H_2_O_2_ and urea are comprehensively reviewed. As compared to NiO and Co_3_O_4_, the NiCo_2_O_4_ nanomaterials showed better electrochemical sensing as adjudged by broader redox peaks with larger area coverage in the CV curves. The biosensing efficiency of the NiCo_2_O_4_ nano-/microstructures can be improved by engineering the morphology, specific surface area, porosity, doping and by making composite/hybrids with various carbonaceous materials, conducting polymers, metal oxides, non-metals and metals. These materials not only improve the mechanical, thermal, and chemical stability but also modulate the bandgap energies, electronic and ionic conductivities, dispersion behavior, avoid aggregation of the NiCo_2_O_4_ nanomaterials and provide short electron and ion diffusion pathways. All these factors contribute to better electrocatalytic behavior with excellent sensitivity, selectivity, and long-term stability of the spinel NiCo_2_O_4_ nano-/microstructure-based biosensors. It is hoped that this review will provide basic ideas as well as new insights for future research and progress in this field.

## Challenges and Future Perspectives

Despite extensive research in this area, many issues that impede the practical application of NiCo_2_O_4_nano-/microstructures need to be addressed for further improvement. Some of these issues have been identified herein.

Structural features of the NiCo_2_O_4_ nanomaterials are controlled by factors like temperature, pH of the reaction solution, precursor concentration, solvent nature and quantity, presence of the growth directing agents and templates, etc. It is, therefore, one of the major challenges to design large-scale and low-cost morphology controlled synthesis of the NiCo_2_O_4_ nanomaterials for next-generation biosensors.

Rational combination of NiCo_2_O_4_ nano-/microstructures with other hybrid materials or conductive substrates to designing NiCo_2_O_4_ composite/hybrids is found to improve the intrinsic characteristics like low electronic conductivity and wide bandgap and hence the biosensing behavior of NiCo_2_O_4_. However, still, more in-depth understanding is required to correlate the synergism between the components of the composite/hybrid materials.

Cost-effectiveness, easy to manufacture, recyclability, sensor disposal, and biocompatibility of NiCo_2_O_4_ nano-/microstructure-based biosensors are other issues that need to be addressed and solved. The high cost of electrochemical work stations restricts the practical applications of these sensors. In this regard, portable and wearable sensing devices will be promising. The toxicity issues of the NiCo_2_O_4_ nano-/microstructures and other components are very rarely discussed in the literature. Future research thus should also focus on studying this important issue.

The biosensing behavior of the NiCo_2_O_4_ nano-/microstructure-based sensors is affected by factors like working temperature, pH of the medium, scan rates, and applied potential. The optimization of these parameters is rarely addressed. Studying the specificity of NiCo_2_O_4_-based sensors from competitive assays requires an appropriate protocol because it is reported that the sensor can detect glucose, H_2_O_2_, urea, trypsin, etc.

Due to complex structures of the biomolecules, the interface and mechanistic studies at the surface of the nanomaterials are still undefined. The redox transformation of the molecules during electrocatalytic biosensing is also a debatable issue. Therefore, future work should focus on elucidating the interaction mechanism between nanomaterials and biomolecules on the electrode surface, to fabricate a new generation of biosensors.
